# Estimation of the applicability domain of kernel-based machine learning models for virtual screening

**DOI:** 10.1186/1758-2946-2-2

**Published:** 2010-03-11

**Authors:** Nikolas Fechner, Andreas Jahn, Georg Hinselmann, Andreas Zell

**Affiliations:** 1 Center for Bioinformatics Tübingen (ZBIT), University of Tübingen, Sand 1, 72076 Tübingen, Germany

## Abstract

**Background:**

The virtual screening of large compound databases is an important application of structural-activity relationship models. Due to the high structural diversity of these data sets, it is impossible for machine learning based QSAR models, which rely on a specific training set, to give reliable results for all compounds. Thus, it is important to consider the subset of the chemical space in which the model is applicable. The approaches to this problem that have been published so far mostly use vectorial descriptor representations to define this domain of applicability of the model. Unfortunately, these cannot be extended easily to structured kernel-based machine learning models. For this reason, we propose three approaches to estimate the domain of applicability of a kernel-based QSAR model.

**Results:**

We evaluated three kernel-based applicability domain estimations using three different structured kernels on three virtual screening tasks. Each experiment consisted of the training of a kernel-based QSAR model using support vector regression and the ranking of a disjoint screening data set according to the predicted activity. For each prediction, the applicability of the model for the respective compound is quantitatively described using a score obtained by an applicability domain formulation. The suitability of the applicability domain estimation is evaluated by comparing the model performance on the subsets of the screening data sets obtained by different thresholds for the applicability scores. This comparison indicates that it is possible to separate the part of the chemspace, in which the model gives reliable predictions, from the part consisting of structures too dissimilar to the training set to apply the model successfully. A closer inspection reveals that the virtual screening performance of the model is considerably improved if half of the molecules, those with the lowest applicability scores, are omitted from the screening.

**Conclusion:**

The proposed applicability domain formulations for kernel-based QSAR models can successfully identify compounds for which no reliable predictions can be expected from the model. The resulting reduction of the search space and the elimination of some of the active compounds should not be considered as a drawback, because the results indicate that, in most cases, these omitted ligands would not be found by the model anyway.

## 1 Background

An important task of cheminformatics and computational chemistry in drug research is to provide methods for the selection of a subset of molecules with certain properties from a large compound database. Often, the desired property is a high affinity to a certain pharmaceutical target protein, and in the selected subset, the likelihood of a compound to be active against that target should be considerably higher than the average in the database. A common approach to this task is virtual screening (VS) [1,2]. The idea is to predict a kind of activity likelihood score, to rank a compound database according to this score and to choose the top ranked molecules as the subset.

A variety of approaches has been published for the assignment of the desired score to a molecule. They can be roughly divided into three classes: Docking-based scoring functions, scores depending on similarity to known active compounds and machine learning-based score predictions.

Docking-based approaches [3-8] rank the compounds according to the score obtained by a docking of the compound into the binding pocket of the respective target protein. Therefore, these approaches use not only the information about the small molecule but also the structure of the target to estimate the activity; however, this additional information comes at the expense of an increased prediction time and the need for a 3D structure of the protein.

The computationally fastest approach to rank the compound database, according to the estimated activity, is to sort the molecules by their similarity to one or more known binders. This approach gives good results in many cases [9-12], but depends strongly on the chosen query molecule and may be unable to discover ligands of a different chemotype than the query molecule [13].

The application of a machine learning model can be considered as a trade-off between a fast prediction time and the integration of additional information. In contrast to the similarity-based ranking, not only information about known active compounds can be used, but also known inactive compounds [14-17]. However, the prediction is based on the prior assumption that the structure-activity relationship is implicitly contained in the training set. Therefore, it is important to be able to decide whether the learned model's prediction of the activity of a molecule should be considered as reliable. In a similarity-based ranking, this decision is not as important, because the similarity score is directly related to the similarity of the activity model represented by the query molecule and the predicted compound. Unfortunately, this direct relation is not present in a learned model that predicts a complex property, like the quantitative activity given as pKi or pIC50, and ranks the compounds according to that property.

In order to address this reliability estimation problem, the concept of the applicability domain (AD) of a machine-learning model has been introduced [18-23]. Usually, it is applied in the context of a quantitative structure-activity relationship (QSAR) application. Here, the task is to predict the activity of a compound, where the AD is used to estimate the relative quantitative prediction error. Nevertheless, it should also be valuable for virtual screening, because due to the large size of the screening data set, it is to be expected that the structural diversity, and thus the impact of an inapplicable model assumption, is greater than that for the structurally more focused QSAR experiments.

Many approaches to estimate the applicability domain of a QSAR model either define the geometric subset of the descriptor space that is spanned by the training set, or infer the multivariate probability distribution of the respective descriptor vectors [20-24]. An alternative approach, similar to the concepts proposed in this work, is to relate the applicability of a model to predict the property of a molecule to the similarity of this compound to the training set structures [18,19]. However, in most cases the models have been trained using a vectorial representation of the molecules, so the vectorial model and the similarity-based applicability domain are defined in different spaces. Nevertheless, these approaches often give convincing results. Recently, Horvath et al. [21] published a meta-model approach to applicability domain estimation and proposed an evaluation procedure for QSAR applicability domains. Unfortunately, these ideas cannot be easily extended to the performance measures for virtual screening used in this work, because they are largely based on the quantitative differences between predictions and observations.

Another concept related to the applicability domain estimation is the estimation of the predictive variance using Gaussian processes [25]. This concept has been successfully applied in QSAR/QSPR [26-28] as well as in virtual screening [29], but has the disadvantage that the error estimation is computationally very demanding.

The definition of the domain of applicability of the training set using the vectorial representation of the training samples by molecular descriptors prevents a direct application to models trained by structured kernel-based machine learning approaches. These machine learning techniques have gained increased attention in recent years in QSAR [30-34] as well as in virtual screening [29,35,36]. Kernel-based learning algorithms, like support vector machines (SVMs) [37,38], infer the model for the relationship between the samples and the respective properties in a high dimensional feature space, which is only implicitly defined by the kernel. A kernel can be considered as a special case of similarity measure fulfilling these additional kernel properties. Many kernels have been published, which cover different aspects of molecular similarity but avoid the loss of information caused by the encoding of the molecule as a descriptor vector. However, the lack of a vectorial representation prevents the direct application of many of the applicability domain formulations.

In this work, we propose three formulations of the applicability domains of kernel-based QSAR models, which rely only on the kernel similarity of the structures, and thus can be applied to assess the reliability of these models. The proposed AD definitions are evaluated quantitatively on three different support vector machine based VS experiments, consisting of screening test data sets based on the DUD repository [39,40] and respective training data sets taken from different sources [41-45]. Furthermore, to avoid a kernel bias, we evaluated three different types of structured molecule kernels: the Optimal Assignment kernel (OAK)[46], the Flexible Optimal Assignment Kernel (FlexOAK)[47] and the Marginalized Graph Kernel (MARG)[48].

We show that, in most cases, the reliability of the ranking produced by the virtual screening is improved and present guidelines for the application of this technique in other experiments.

## 2 Methods

### 2.1 Metrics for Virtual Screening Evaluation

Many performance measures for the evaluation of a prediction model, such as Matthew's correlation coefficient, accuracy, or precision, are unsuitable metrics for the evaluation of virtual screening experiments. The reason for this unsuitability is that these metrics do not address the early recognition problem. The early recognition problem is motivated by real-world VS experiments, where only the top ranked compounds of the complete data set are evaluated in biological assays. To address this problem, several virtual screening metrics were suggested and applied to compare different VS techniques. One of those metrics is the Enrichment Factor, which is defined for a predefined subset of the data set. This measure indicates the ratio of the probabilities of finding an active compound in the first X% of the data set and that of sampling one at random from the complete data set. Despite its early recognition sensitivity, the Enrichment Factor has the drawback of being insensitive to the relative ranking of the compounds in the top X% and ignoring the complete ranking of the remaining data set [49]. To address this problem, Truchon and Bayly [50] developed a ranking sensitive metric with a modifiable focus on the early recognition problem, the BEDROC score. This metric uses an exponential decay function to reduce the influence of lower ranked compounds on the final score. The score has a parameter α that allows the user to adjust the definition of the early recognition problem. In this work, four different α values were used to define different weightings of the top ranked structures. Table 1 shows the α values applied, together with the section of the data set that is responsible for 80% of the final BEDROC score.

**Table 1:** Relation between α and the fraction of the screening set contributing 80% of the BEDROC score

Data set percentage	α-value
1.6%	100.0
3.0%	53.6
3.2%	50.0
5.0%	32.2

According to Table 1, an α value of 100.0 parameterizes the BEDROC score such that the ranking in the first 1.6% of the data set contributes 80% of the final BEDROC score.

### 2.2 Kernel-based Machine Learning

Kernel-based machine learning techniques, like support vector machines, have become a common approach to learning quantitative structure-activity relationships. In contrast to other machine learning algorithms, like partial least squares or neural networks, kernel-based models do not need an explicit feature encoding *φ *(*x*) (i.e., a descriptor representation) for an object *x *(e.g., a molecule) from the data space *χ*. Instead, the data objects only occur in terms of dot products ⟨*φ *(x), *φ *(x')⟩ between pairs of descriptor representations, which allow the so-called kernel trick to be applied. Here, the idea is to replace both, the feature encoding *φ *and the dot product by one function *k*: *χ *× *χ *→ ℜ, which directly maps a pair of objects to a real number. The function *k *can be any kernel function for which there exists a (not necessary explicit) descriptor encoding function *φ*: *χ *→ ℜ^*n*^, such that the kernel of two objects *x*, *x*' corresponds to the dot product of their respective descriptor representations *φ *(x), *φ *(x'):(1)

This condition is fulfilled by any kernel function that is symmetric and positive semi-definite. Thus, the feature encoding *φ *(*x*) does not have to be stated explicitly and the kernel can be formulated directly on the data object (e.g., the molecule). Therefore, the necessity to encode a chemical structure as a numerical vector using a set of explicitly calculated molecular descriptors could be avoided by using a structured kernel (i.e., a kernel that takes non-numerical objects). In this case, the kernel method still operates in a vectorial space, but the numerical representations of the data objects in that space never have to be computed explicitly.

This article can only give a short introduction to the idea of kernel-based machine learning. An excellent overview as well as detailed descriptions of various kernel-based techniques can be found in Schölkopf and Smola [51] for further reading.

### 2.3 Structured Kernels for Molecules

Structured kernels for complex objects usually resemble sensible similarity measures for these types of objects. In recent years, several such kernels have been published that directly operate on molecular graphs and thus avoid an explicit descriptor representation of the molecules. The structured kernels that are used in this work are the Marginalized Graph kernel (MARG) [48], the Optimal Assignment kernel (OAK) [46], and its extension FlexOAK [47], which regards the molecular flexibility. Other examples are the iterative Optimal Assignment approach [33], the Pharmocophore kernel [35] and several other variants of molecular fingerprint-like formulations [32,34,36].

The kernels that are used in this work describe the similarity between two molecules by considering pairwise atomic similarities and combining these in different ways to compare the molecular structure. The atoms are encoded by a set of atom descriptors consisting of both nominal (e.g., part of ring, part of HB-donor, ...) and numeric (e.g., Gasteiger-Marsili partial charges [52], graph potentials [53], ...) features. The complete list of the applied atom descriptors (as well of the bond descriptors) is given in the additional file 1. The resulting feature representation of the atoms is used to define the atomic similarity between two atoms. All kernels use the same atomic similarity measure, but integrate the single pairwise atomic similarities in different ways. The atom and bond descriptors applied in this work were computed using the open-source Java cheminformatics library JOELib2 [54]. To conduct QSAR experiments and to generate the QSAR models used for the virtual screening in this work, the structured kernels presented are used as kernels in a support vector regression. Hence, the molecular representation, on which the QSAR is computed, is solely given as the implicit feature encoding hidden in the structured kernel. Thus, in contrast to most other QSAR related works, there is no need for a set of molecular descriptors, beside the atom and bond descriptors used by the kernels, to encode the molecules as numerical vectors.

The Marginalized Graph kernel (MARG) was published by Kashima et al. [48] in 2003. Its idea is to define the similarity of two molecules as the sum of similarities of atom sequences present in the molecules. This concept is similar to various hashed fingerprint formulations (e.g., Daylight) but has some differences. The Marginalized Graph kernel considers every atom sequence of arbitrary length in the molecule. Atoms are allowed to occur multiple times in the sequences, thus rings may lead to infinitely long sequences. The similarity of two atom sequences is given as the sum of the atom similarities of the pairs of atoms in corresponding positions if the sequences have the same length, and is set to zero otherwise. The final kernel is obtained by summing the similarities of the atom sequences of the molecules, whereas each sequence similarity is additionally weighted by the probability that the respective sequence is randomly drawn from the set of possible sequences in the molecule. The multiplication by the probability reduces the contribution of longer sequences because they have an obviously lower probability of being drawn randomly. This decreased contribution of long sequences has the effect that the weighted sum converges to a limit. This limit can be computed without explicitly extracting the atom sequences from the molecules by exploiting the product graph of the molecular graphs. Therefore, the Marginalized Graph kernel is able to consider an infinite number of possible atom sequences by computing the limit of the weighted sum of the similarities directly. The obtained limit is the expectation of the sequence set similarity and is taken as the kernel value. The details of the algorithm are beyond the scope of this article and are therefore omitted.

The Optimal Assignment kernel published by Fröhlich et al [46] computes the kernel similarity of two molecules by calculating the optimal bipartite graph matching of the atoms of the two molecules. Each molecule is regarded as a set of atoms augmented by their local intramolecular neighborhoods. The kernel is obtained by computing the optimal pairwise assignment of the atoms (including their intramolecular neighboring atoms) of the first molecule to the atoms of the second molecule. The final kernel value is obtained as the sum of the similarities of the assigned atoms normalized by the self-similarities (i.e., the kernel similarities) of the respective molecules. The normalization ensures that the kernel values are always in the interval [0,1]. A kernel value of one is only obtained for identical (by means of the kernel) molecules.

The FlexOAK [47] has been published recently as an extension of the OAK capable of regarding the intramolecular flexibility of the molecules. Its idea is to introduce a further measure for atom similarity that takes the flexibility of the local intramolecular neighborhood of the compared atoms into account. This extended local similarity computation is realized by comparing the spatial coordinates, on which the neighboring atoms (up to neighbors of degree three, i.e., connected by sequences of up to three bonds) can be placed by rotating some of the connecting bonds. The spatial coordinates are encoded by a set of translation and scale invariant parameters related to internal coordinates. The comparison is then conducted by computing a numerical similarity of the parameter sets of the two compared atoms. Thus, the similarity of the local flexibilities of the two atoms is obtained as the similarity of the parameters of the spatial positions and incorporated into the optimal assignment computation. All other similarity aspects (i.e., structural similarity and atomic descriptors) are identical to the original OAK.

It has been shown [55] that kernels that are based on an optimal assignment are not in general positive semi-definite (i.e., their pairwise kernel-similarity matrix may have negative eigenvalues). Nevertheless, the negative eigenvalues typically observed in in-house experiments, are so close to zero (usually > -0.0001) that it should not have an effect on the practical application. This conclusion can be drawn because, due to numerical inaccuracies, kernels that have been proven to be positive semi-definite, sometimes yield similar small eigenvalues.

The source code of the Optimal Assignment and the Marginalized Graph kernel implementations can be obtained from our department's homepage [56]. A working JOELib2 [54] environment is necessary for their application.

### 2.4 Applicability Domain Estimation for Kernel Machines

The applicability domain of a statistical model is defined as the subset of the underlying pattern space (e.g., the ChemSpace) on which the model is expected to give reliable predictions [19,20,22-24]. This formulation makes the adaptation of this concept for kernel-based learning methods difficult. In contrast to non-kernel techniques, like most neural networks or decision trees, the kernel model performs its estimations based on the structure of the training set in the feature space implicitly defined by the kernel. Therefore, the applicability domain has also to be defined by means of the kernel. Because some kernels, like the RBF kernel, rely on a descriptor vector, and the applicability domain of the training set could be approximated in these cases by geometrical approaches, in this work the selection of the kernels was restricted to structured kernels, which work directly on the molecular graph.

A possible kernel-based approach would be the application of a Gaussian process to learn the model and exploit its possibility to augment a prediction with its estimated standard deviation. However, despite its similarity to the above methods, this approach has to be regarded more as a confidence estimation than as an applicability domain estimation. Moreover, a Gaussian process has the drawback that it requires a full matrix inversion of the covariance matrix for each confidence estimation (it can be approximated, but the complexity is still larger than quadratic, see [57] for details), which is computationally more demanding than the actual prediction.

In this work, three pure kernel-based descriptions of the applicability domain are introduced: the calculation of the subspace of the implicit feature space that contains most of the training patterns, which can be learned with a one-class support vector machine and two approaches to defining the domain boundary by a threshold for the (weighted) average kernel similarity. In contrast to the Gaussian process confidence estimation, the proposed AD estimations can be calculated with almost no computational overhead for the prediction.

#### 2.4.1 Kernel Density Estimation

The Kernel Density Estimation (KDE) is a non-parametric approach to estimate the probability density of a distribution given a set of *n *samples drawn from this distribution and a kernel function *k*. The Gaussian kernel density of a pattern set *X *at *x *is given by:(2)

The smoothing parameter *h *could be fitted to the application, but is neglected in our formulation to ensure compatibility to an arbitrary kernel.

This concept can be adapted to other kernels by replacing the Gaussian part by the kernel(3)

This density is identical to the average kernel similarity of *x *to the patterns *x*_*i *_from the training set *X*. Therefore, this approach is closely related to the similarity-based AD formulations [18,19], with the difference that the formulation in this case ensures that the AD and the model are both defined in the same kernel-induced feature space. Thus, it should be a good estimate of the information that a kernel-based model has about that part of the feature space or, by probabilistic means, how likely it is that the sample *x *was drawn from the same distribution as the training set.

#### 2.4.2 Weighted Kernel Density Estimation

The kernel density estimate can be further augmented with the knowledge that the model (in this case a support vector machine) was obtained during its training by applying an approach motivated by a Gaussian mixture model. A Gaussian mixture model is a model of a complex probability distribution described as a weighted sum of Gaussian distributions with means *x*_*i *_and variances . In the one-dimensional case, this model is expressed by:(4)

The model parameters that are adapted to the observation are the mixing coefficients α_*i *_and the means and standard deviations of the Gaussians. This concept serves as the starting point to define a model-dependent kernel-based applicability domain estimation. The key idea is to integrate the knowledge encoded in a kernel model into a Gaussian mixture scheme. This integration is achieved by regarding the support vectors of the SVM model as the centers of Gaussian distributions whose (co)variances are represented by the applied kernel. The mixing coefficients of the Gaussian mixture model are replaced by the weights of the support vectors (i.e., the signed Lagrange coefficients) that represent the learned knowledge of the trained model. To obtain a probability density for the distribution related to a support vector, the Gaussian density is expressed by means of the kernel. To achieve this expression, several simplifications are introduced. First, only 1-dimensional probability densities are considered because the density is always only evaluated for a single test sample, which allows a 1-dimensional embedding of the feature space defined by the kernel and two data points (the support vector and the test sample). Second, the Lagrange coefficients that replaced the mixing coefficients are in general not valid probabilities. Therefore, the modified mixture model cannot be considered as a probability density. However, this apparent drawback allows us to neglect the normalization term of the kernel-based 1D Gaussian probability density. Thus, the latter can be obtained using the identity of the kernel value for an object pair (*x*, *y*) ∈ *X *× *X *and the inner product of the respective feature space projection *φ *(*x*) for *x *∈ *X*:(5)

Using this identity, the Gaussian distributions with means *μ*_*i *_and variances  can be expressed by means of the kernel:(6)

Note that the kernel is assumed to be normalized (i.e., returns 1 if the two objects are identical). This assumption is always met by the kernels used in this work, and can be easily ensured for a general kernel by the following normalization:(7)

Thus, the final confidence estimation can be completely expressed by means of the kernel:(8)

The variance of the single Gaussian terms can be adapted to the problem, but is kept fixed to one in the implementation used in this work. One drawback of this formulation is that the score does depend on the number of support vectors as well as on the absolute value of the Lagrange coefficients. To address this problem the score obtained by Eq. (7) is normalized:(9)

#### 2.4.3 One Class SVM

The One-Class SVM, published by Schölkopf et al. [58], is an approach to compute the domain of a set of *n *training samples in the kernel induced feature space. The result is a decision function, which returns a positive value for a data instance inside the domain and a negative for those outside of it. Like standard SVMs it is formulated as a quadratic program in which the data from an arbitrary space χ only appears in an inner product in the dual formulation and thus can be replaced by any Mercer kernel *k*: χ × χ → ℜ. The optimization problem is given by:(10)

The regularization can be adapted by the parameter ν, which can be regarded as an upper bound on the fraction of training set outliers [38]. The One-Class SVM can be applied in the applicability domain estimation by training a One-Class model on the training data set and using the value of the decision function as an applicability score.(11)

Note that the model selection is more difficult than that in regression or classification because no quantitative quality measure can be optimized with a cross-validation on the training data. Therefore, the regularization parameter has to be chosen solely by the user. It this work, this parameter has been kept constant using the LibSVM 2.83 [59] default settings.

## 3 Experimental

### 3.1 Data Sets

The evaluation of the proposed methods for the applicability domain estimation has requirements that restrict the number of freely available data sets. An established resource of data sets for VS experiments is the DUD collection published by Huang et al. [39]. Originally designed for docking studies, these data sets have recently been prepared for ligand-based virtual screening experiments by Jahn et al. [10,40] according to the preparation protocol proposed by Cheeseright et al. [60] and Good and Oprea [61]. A subset of the data sets prepared by Jahn et al [10,40] was chosen to provide as external screening test sets for the quantitative evaluation of the applicability domain estimation. The subset was selected according to two criteria. First, there has to be another data set containing enough molecules with known quantitative activities to the respective target to serve as a training set for a QSAR model. Second, no molecule from the training set may be part of the screening set. The use of a procedure for a removal of duplicates was rejected because the active/decoy ratio is constant for all DUD data sets and the removal of the duplicates from the training set would result in a huge loss of training samples. This loss is, because in many observed cases (e.g. the Sutherland compilation [45]) a large portion or even the complete potential QSAR training set was incorporated in the DUD data set.

These requirements were met for Thrombin, Factor Xa and Platelet-derived growth factor receptor β (PDGFRβ) (Table 2). The screening sets were taken from Jahn et al. [10,40] and consisted of 24 actives and 1148 decoys for Thrombin, 64 actives and 2092 decoys for Factor Xa and 124 actives and 5603 decoys for PDGFRβ. The respective training sets were taken from Sutherland et al. [45] (88 molecules annotated with pK_i _Thrombin inhibition, originally published by Böhm et al. [41]), Fontaine [42] (290 molecules annotated with pK_i _Factor Xa inhibition) and Guha [43] (79 molecules annotated with pIC50 PDGFR-β inhibition, originally published by Pandey et al. [44]).

**Table 2:** Statistics of the data sets for the different targets

Target	Training Compounds	Screening Compounds	Ligands	Decoys
Thrombin	88	1172	24	1148
Factor Xa	290	2156	64	2092
PDGFRβ	79	5727	124	5603

The disjunction of the training and the screening sets was checked by comparing the unique SMILES representations of the molecules. Because the kernels used in this work are not able to differentiate between stereoisomers, the stereochemical information in the SMILES was neglected. Thus, stereoisomers would have been considered as identical if there would have had been any in the data sets.

All datasets used in this work are publicly available. The exact SD files [62] used for training can be obtained from our department's website [63]. The screening datasets can be obtained from the DUD site listed as *DUD LIB VS 1.0 *[64].

### 3.2 QSAR learning and virtual screening

A single experimental setup is determined by the target protein to be modeled, the structured kernel, which is used for SVM and applicability calculation, and the formulation of the applicability domain. Each experiment was conducted in the same fashion. A standard QSAR model was learned on the training data set using ε-support vector regression and the respective kernel. The only parameter that was optimized was the soft margin SVM parameter C. The optimization was conducted by searching for the value for *C *in the set {e^-5^, e^-4^,..., e^4^, e^5^} that minimizes the average RMSE on a 5-fold cross-validation. The cross-validation estimates of the model performance are shown in Table 3. The model selection that yields these estimates is biased to the selection of the cross-validation folds, so the results cannot be considered as reliable estimates of the true model performance. However, they give a hint of the descriptive power of the model for the training set, and the main results of this work on the screening data sets are obtained independently of this training set performance estimation. The training of the SVM was conducted using the Java implementation of LibSVM 2.83.

**Table 3:** Results of the QSAR training and the virtual screening without consideration of the applicability domain

	Thrombin			Factor Xa			PDGFRβ		
	**OAK**	**FlexOAK**	**MARG**	**OAK**	**FlexOAK**	**MARG**	**OAK**	**FlexOAK**	**MARG**

	**QSAR Training**								

Q^2^	0.49 ± 0.17	0.52 ± 0.15	0.52 ± 0.11	0.82 ± 0.08	0.85 ± 0.06	0.78 ± 0.1	0.38 ± 0.24	0.37 ± 0.29	0.40 ± 0.22
RMSE	0.55 ± 0.22	0.51 ± 0.15	0.50 ± 0.17	0.63 ± 0.24	0.53 ± 0.17	0.77 ± 0.29	0.34 ± 0.25	0.38 ± 0.30	0.32 ± 0.22

	Virtual Screening without Applicability Domain Estimation								

AUC	0.45	0.51	0.54	0.74	0.68	0.54	0.58	0.54	0.25
BEDROC (100.0)	0.42	0.29	0.29	0.32	0.28	0.00	0.13	0.10	0.07
BEDROC (53.6)	0.37	0.29	0.27	0.31	0.25	0.01	0.12	0.09	0.06
BEDROC (32.2)	0.37	0.29	0.27	0.33	0.24	0.01	0.12	0.09	0.06
BEDROC (20.0)	0.35	0.30	0.28	0.37	0.26	0.02	0.13	0.11	0.05

**Figure 1:**
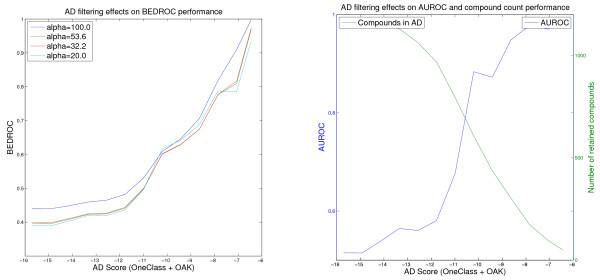
**Effect of the One-Class AD on the Thrombin VS performance of the OAK model**. Virtual screening of the Thrombin data set using the Optimal Assignment kernel and the One Class SVM AD Formulation (values for the threshold retaining less than 50 compounds in the AD are omitted).

**Figure 2:**
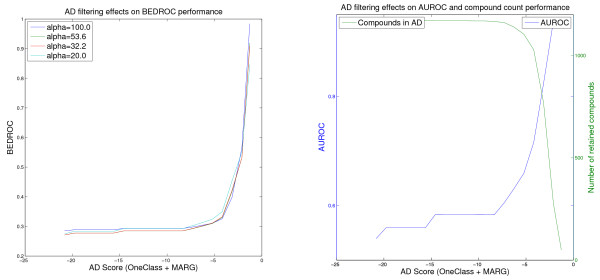
**Effect of the One-Class AD on the Thrombin VS performance of the MARG model**. Virtual screening of the Thrombin data set using the Marginalized Graph kernel and the One Class SVM AD Formulation (values for the threshold retaining less than 50 compounds in the AD are omitted).

**Figure 3:**
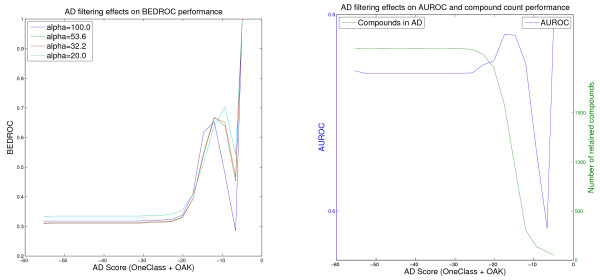
**Effect of the One-Class AD on the Factor Xa VS performance of the OAK model**. Virtual screening of the Factor Xa data set using the Optimal Assignment kernel and the One Class SVM AD Formulation (values for the threshold retaining less than 50 compounds in the AD are omitted).

**Figure 4:**
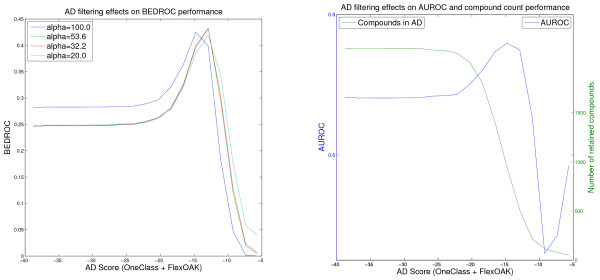
**Effect of the One-Class AD on the Factor Xa VS performance of the FlexOAK model**. Virtual screening of the Factor Xa data set using the FlexOAK kernel and the One Class SVM AD Formulation (values for the threshold retaining less than 50 compounds in the AD are omitted).

**Figure 5:**
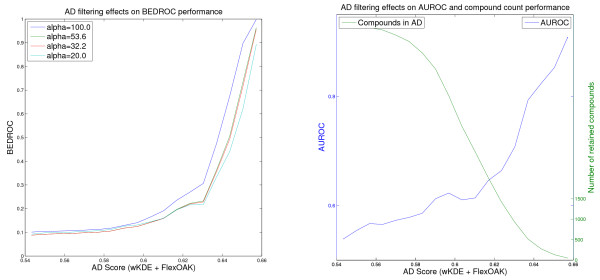
**Effect of the wKDE AD on the PDGFRβ VS performance of the FlexOAK model**. Virtual screening of the PDGFRβ data set using the Flexible Optimal Assignment kernel weighted KDE AD Formulation (values for the threshold retaining less than 50 compounds in the AD are omitted).

**Figure 6:**
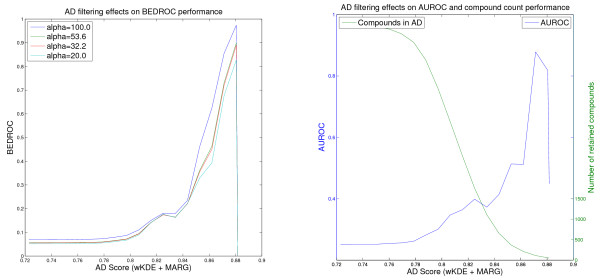
**Effect of the wKDE AD on the PDGFRβ VS performance of the MARG model**. Virtual screening of the PDGFRβ data set using the Marginalized Graph kernel and weighted KDE AD Formulation (values for the threshold retaining less than 50 compounds in the AD are omitted).

For the screening evaluation, the learned model is used to rank the respective screening data set according to the learned property (i.e. pK_i _or pIC50). The different activity measures can be neglected because they are consistent for each experiment (Thrombin, Factor Xa, PDGFRβ) and for the screening evaluation only the ranking of the compound and not the exact activity is of importance. For each screening data set, the rankings are further filtered using one of the three applicability score formulations. The complete range of scores on the screening set is divided into 20 equidistant score thresholds in Figures 1, 2, 3, 4, 5 and 6. In these figures, the score threshold values, for which less than 50 compounds are retained in the AD, are omitted, because the performance measures are not robust for such small data sets. Otherwise, the evaluation of these small data sets would result in large changes in the performances caused by small changes in the screening ranking (i.e., with less than 50 compounds retained a change of the top ranked compound alone would affect 80% of the BEDROC(100) score). In addition to the six example figures, a complete list of the figures for the different experiments is presented in the additional file 2.

In addition, the thresholds are determined, which retain 50%, 33%, exactly 200, and exactly 100 compounds in the AD to provide comparable performance measures (Tables 4, 5 and 6). For each score threshold, the screening data set is divided into a set of molecules that possess an AD score lower than the threshold and thus are considered as outliers, and a set of molecules for which the prediction is regarded as reliable at the respective applicability level.

**Table 4:** Virtual Screening results for Thrombin at five different applicability levels

Kernel	ADE		Threshold	AUROC	BEDROC (α)				Ligands	Decoys
					100.00	53.6	32.2	20.0		
OAK	KDE	50%	0.64	**0.88**	**0.61**	**0.60**	**0.60**	**0.61**	11	575
		33%	0.66	**0.87**	**0.66**	**0.63**	**0.63**	**0.64**	10	380
		200	0.68	**0.97**	**0.78**	**0.72**	**0.73**	**0.73**	8	192
		100	0.69	**0.97**	**0.90**	**0.81**	**0.81**	**0.78**	8	92
	wKDE	50%	0.64	**0.88**	**0.61**	**0.60**	**0.60**	**0.61**	11	575
		33%	0.66	**0.87**	**0.66**	**0.63**	**0.63**	**0.64**	10	380
		200	0.68	**0.97**	**0.78**	**0.73**	**0.73**	**0.74**	8	192
		100	0.69	**0.97**	**0.90**	**0.81**	**0.81**	**0.78**	8	92
	ONE	50%	-9.61	**0.88**	**0.62**	**0.60**	**0.60**	**0.60**	11	478
		33%	-9.11	**0.87**	**0.66**	**0.63**	**0.63**	**0.64**	10	380
		200	-8.06	**0.94**	**0.77**	**0.69**	**0.68**	**0.68**	9	191
		100	-7.08	**0.97**	**0.90**	**0.81**	**0.81**	**0.78**	8	92

FlexOAK	KDE	50%	0.64	**0.85**	**0.43**	**0.47**	**0.48**	**0.52**	11	575
		33%	0.65	**0.91**	**0.49**	**0.53**	**0.53**	**0.59**	9	381
		200	0.67	**0.91**	**0.59**	**0.58**	**0.58**	**0.63**	8	192
		100	0.68	**0.91**	**0.74**	**0.67**	**0.67**	**0.69**	8	92
	wKDE	50%	0.64	**0.78**	**0.41**	**0.45**	**0.46**	**0.49**	12	574
		33%	0.65	**0.91**	**0.49**	**0.53**	**0.53**	**0.59**	9	381
		200	0.67	**0.91**	**0.59**	**0.58**	**0.58**	**0.63**	8	192
		100	0.68	**0.91**	**0.74**	**0.67**	**0.67**	**0.69**	8	92
	ONE	50%	-9.80	**0.91**	**0.49**	**0.54**	**0.54**	**0.59**	10	576
		33%	-9.07	**0.91**	**0.55**	**0.59**	**0.60**	**0.64**	9	381
		200	-8.10	**0.92**	**0.62**	**0.63**	**0.64**	**0.68**	8	192
		100	-7.32	**0.98**	**0.74**	**0.68**	**0.68**	**0.71**	7	93

MARG	KDE	50%	0.876	**0.81**	**0.45**	**0.43**	**0.43**	**0.45**	12	574
		33%	0.885	**0.96**	**0.56**	**0.58**	**0.58**	**0.62**	8	382
		200	0.893	**0.94**	**0.62**	**0.58**	**0.58**	**0.59**	8	192
		100	0.899	**0.93**	**0.90**	**0.80**	**0.79**	**0.74**	8	92
	wKDE	50%	0.882	**0.81**	**0.46**	**0.44**	**0.44**	**0.47**	12	574
		33%	0.891	**0.96**	**0.56**	**0.58**	**0.58**	**0.62**	8	382
		200	0.899	**0.95**	**0.77**	**0.69**	**0.69**	**0.68**	8	192
		100	0.905	**0.93**	**0.90**	**0.80**	**0.79**	**0.74**	8	92
	ONE	50%	-2.631	**0.80**	**0.42**	**0.42**	**0.43**	**0.46**	11	531
		33%	-2.334	**0.88**	**0.52**	**0.50**	**0.50**	**0.52**	9	381
		200	-1.932	**0.93**	**0.61**	**0.54**	**0.54**	**0.54**	8	192
		100	-1.607	**0.92**	**0.73**	**0.64**	**0.63**	**0.61**	7	93

The threshold is adjusted such that either a certain fraction (50%, 33%) of the compounds or that exactly n compounds (*n *= 200, 100) remain in the domain. Results, which differ significantly from random rankings (*p*-Value < 0.01) are shown in bold.

**Table 5:** Virtual Screening results for Factor Xa at five different applicability levels

Kernel	ADE		Threshold	AUROC	BEDROC (α)				Ligands	Decoys
					100.00	53.6	32.2	20.0		
OAK	KDE	50%	0.645	**0.78**	**0.59**	**0.52**	**0.51**	**0.49**	58	1020
		33%	0.65	**0.78**	**0.68**	**0.62**	**0.61**	**0.57**	57	661
		200	0.67	**0.74**	0.58	**0.68**	**0.69**	**0.69**	52	148
		100	0.68	0.62	0.36	0.53	0.54	0.61	40	60
	wKDE	50%	0.65	**0.78**	**0.59**	**0.52**	**0.51**	**0.49**	58	1020
		33%	0.66	**0.78**	**0.65**	**0.59**	**0.58**	**0.55**	57	661
		200	0.67	**0.74**	0.58	**0.68**	**0.69**	**0.69**	53	147
		100	0.68	0.62	0.36	0.55	0.58	0.64	42	58
	ONE	50%	-15.09	**0.78**	**0.60**	**0.53**	**0.53**	**0.50**	58	966
		33%	-13.98	**0.78**	**0.65**	**0.58**	**0.51**	**0.53**	58	660
		200	-10.84	**0.73**	0.58	**0.68**	**0.69**	**0.69**	56	144
		100	-7.33	0.61	0.36	0.55	0.57	0.64	45	55

FlexOAK	KDE	50%	0.64	**0.76**	**0.42**	**0.38**	**0.38**	**0.37**	59	1019
		33%	0.64	**0.75**	**0.43**	**0.41**	**0.41**	**0.39**	58	660
		200	0.66	**0.62**	0.11	0.18	0.18	0.22	47	153
		100	0.67	0.48	0.00	0.03	0.04	0.07	43	57
	wKDE	50%	0.64	**0.76**	**0.40**	**0.37**	**0.37**	**0.36**	59	1019
		33%	0.65	**0.74**	**0.40**	**0.39**	**0.38**	**0.37**	57	661
		200	0.66	**0.63**	0.11	0.18	0.18	0.22	48	153
		100	0.67	0.48	0.00	0.03	0.03	0.07	43	57
	ONE	50%	-15.40	**0.76**	**0.41**	**0.38**	**0.38**	**0.37**	59	1016
		33%	-13.86	**0.75**	**0.43**	**0.42**	**0.42**	**0.40**	59	659
		200	-10.92	**0.64**	0.13	0.23	0.24	0.29	51	149
		100	-8.20	0.42	0.00	0.03	0.04	0.08	44	56

MARG	KDE	50%	0.84	0.57	0.00	0.02	0.02	0.3	54	1024
		33%	0.85	0.57	0.00	0.01	0.02	0.03	44	674
		200	0.864	0.48	0.00	0.00	0.00	0.00	21	179
		100	0.866	0.56	0.00	0.00	0.00	0.00	16	84
	wKDE	50%	0.845	0.57	0.00	0.01	0.02	0.03	54	1024
		33%	0.852	0.56	0.00	0.00	0.00	0.01	43	675
		200	0.86	0.47	0.00	0.00	0.00	0.00	21	180
		100	0.87	0.55	0.00	0.00	0.00	0.00	16	84
	ONE	50%	-11.30	0.56	0.01	0.02	0.02	0.03	55	1014
		33%	-9.95	0.55	0.04	0.01	0.01	0.02	43	675
		200	-7.79	0.41	0.00	0.00	0.00	0.00	14	186
		100	-7.09	0.47	0.00	0.00	0.00	0.00	8	92

The threshold is adjusted such that either a certain fraction (50%, 33%) of the compounds or that exactly n compounds (*n *= 200, 100) remain in the domain. Results, which differ significantly from random rankings (*p*-Value < 0.01) are shown in bold.

**Table 6:** Virtual Screening results for PDGFRβ at five different applicability levels

Kernel	ADE		Threshold	AUROC	BEDROC (α)				Ligands	Decoys
					100.00	53.6	32.2	20.0		
OAK	KDE	50%	0.62	**0.68**	**0.22**	**0.19**	**0.19**	**0.19**	74	2789
		33%	0.63	**0.67**	**0.28**	**0.22**	**0.22**	**0.21**	55	1854
		200	0.667	**0.78**	**0.80**	**0.65**	**0.64**	**0.57**	7	193
		100	0.673	**0.79**	**0.95**	**0.83**	**0.82**	**0.72**	6	94
	wKDE	50%	0.62	**0.68**	**0.22**	**0.19**	**0.18**	**0.19**	74	2790
		33%	0.63	**0.67**	**0.28**	**0.22**	**0.22**	**0.21**	56	1853
		200	0.665	**0.76**	**0.79**	**0.63**	**0.61**	**0.53**	8	192
		100	0.672	**0.87**	**0.95**	**0.83**	**0.82**	**0.73**	6	94
	ONE	50%	-11.58	**0.67**	**0.22**	**0.19**	**0.19**	**0.19**	76	2781
		33%	-10.95	**0.66**	**0.26**	**0.20**	**0.20**	**0.19**	58	1851
		200	-8.93	**0.78**	**0.80**	**0.65**	**0.64**	**0.57**	7	192
		100	-8.64	**0.78**	**0.95**	**0.83**	**0.82**	**0.72**	6	94

FlexOAK	KDE	50%	0.61	**0.61**	**0.18**	**0.15**	**0.15**	**0.15**	67	2796
		33%	0.62	**0.65**	**0.25**	**0.21**	**0.20**	**0.20**	52	1857
		200	0.647	**0.84**	**0.79**	**0.61**	**0.60**	**0.53**	9	191
		100	0.653	**0.87**	**0.95**	**0.82**	**0.80**	**0.70**	7	93
	wKDE	50%	0.61	**0.61**	**0.18**	**0.15**	**0.15**	**0.15**	61	2796
		33%	0.62	**0.65**	**0.25**	**0.20**	**0.20**	**0.20**	52	1857
		200	0.646	**0.83**	**0.79**	**0.61**	**0.60**	**0.52**	9	191
		100	0.653	**0.85**	**0.95**	**0.81**	**0.79**	**0.68**	8	92
	ONE	50%	-12.67	**0.61**	**0.18**	**0.15**	**0.15**	**0.15**	67	2796
		33%	-12.04	**0.65**	**0.25**	**0.20**	**0.20**	**0.20**	50	1859
		200	-10.33	**0.82**	**0.79**	**0.61**	**0.59**	**0.52**	9	191
		100	-9.97	**0.86**	**0.95**	**0.82**	**0.80**	**0.70**	7	93

MARG	KDE	50%	0.83	0.37	**0.13**	**0.11**	**0.11**	**0.11**	52	2812
		33%	0.84	0.40	**0.18**	**0.17**	**0.17**	**0.17**	25	1884
		200	0.87	0.54	**0.65**	**0.49**	**0.48**	**0.43**	8	192
		100	0.88	0.76	**0.88**	**0.75**	**0.73**	**0.66**	4	96
	wKDE	50%	0.81	0.35	**0.13**	**0.12**	**0.11**	**0.11**	49	2814
		33%	0.82	0.38	**0.17**	**0.17**	**0.17**	**0.17**	29	1880
		200	0.86	0.57	**0.65**	**0.49**	**0.48**	**0.42**	8	192
		100	0.87	0.86	**0.91**	**0.82**	**0.81**	**0.77**	3	97
	ONE	50%	-5.05	0.39	**0.13**	**0.11**	**0.11**	**0.11**	51	2812
		33%	-4.58	0.42	**0.19**	**0.18**	**0.18**	**0.18**	26	1883
		200	-3.09	0.54	**0.65**	**0.49**	**0.48**	**0.43**	8	192
		100	-2.79	0.85	**0.91**	**0.82**	**0.81**	**0.77**	3	97

The threshold is adjusted such that either a certain fraction (50%, 33%) of the compounds or that exactly n compounds (*n *= 200, 100) remain in the domain. Results, which differ significantly from random rankings (*p*-Value < 0.01) are shown in bold.

## 4 Results and Discussion

The QSAR results for all kernels and data sets are presented in Table 3. The training data sets were not all equally well learned as shown by the different cross-validation results. All kernels were capable of describing the Factor Xa inhibitors in a manner that allows the learning of a QSAR model with a good cross-validation performance. Thrombin and PDGFRβ seem to be less suited for learning the respective QSAR, but despite the low correlation coefficients, the prediction error still was small enough to apply the model in a VS experiment.

The second part of Table 3 shows the virtual screening performance of the QSAR models by means of the Area-under-the-ROC-Curve (AUROC) and the BEDROC scores for four different choices of α (shown in brackets). Except for the AUROC for the Factor Xa data set using either the OAK or the FlexOAK, none of the performance criteria were good enough to expect a successful application of the models for virtual screening.

Generally, the molecules for which wrong predictions are obtained from a model generated using a supervised machine learning algorithm can be roughly divided into two classes: first, molecules that are too dissimilar to the compounds from the training set for us to expect to draw conclusions about them considering only the training set, and second, those molecules that are similar to the molecules from the training set but whose properties are not consistent with the QSAR similarity paradigm [65] that states that similar molecules should have similar properties. The first class should be recognizable using an applicability domain estimation, and should not properly be considered as errors, but rather as outliers, for which the model is not defined. The second class can be considered as "real" errors of the model. A possible cause for such errors may be that the respective molecules come from a part of the ChemSpace that lies inside the applicability domain of the training data set, but has too few training samples to describe the curvature of the modeled property adequately. An alternative possibility is that the modeled property changes rapidly in that part of the space, as would be the case for a so-called activity cliff [66-68]. In both cases, an applicability domain estimation cannot be expected to detect and remove these molecules.

The modest performance of the QSAR models in the virtual screening is the result of unreliable predictions for molecules, for which the models are apparently not specified (i.e., the first type of misprediction), as well as wrong implications learned by the model (i.e., the second type of misprediction). The applicability domain estimation should be able to remove the first kind and to improve the reliability of the predictions accordingly.

### 4.1 Effect on the prediction time

The applicability score using one of the two density-based formulations can be calculated in the same iteration as the model prediction, leading to almost no computation time overhead. In contrast to that, for the One-Class AD estimation a second model has to be generated in addition to the screening model to describe its applicability domain. Thus, for each compound two predictions have to be calculated (one for the screening and one for the AD score) leading to an approximately doubled computation time.

### 4. 2 Thrombin

Table 4 shows several results of the virtual screening experiments for Thrombin. It can be seen that the screening performance of a model is improved as the applicability domain becomes more restrictive. If the threshold is chosen such that exactly 100 compounds remain in the domain, a very good and significant enrichment can be achieved (OAK + One-Class ADE) yielding an AUROC of 0.97 and BEDROC scores between 0.78 and 0.90. The number of ligands is reduced to about one third, but simultaneously the number of decoys is reduced to 3.7%, changing the prior probability of choosing a ligand by chance from 1:36 to ~1:7. The higher random probability of getting a good enrichment is considered by a permutation significance test. The results indicate that all three kernels, as well as all three applicability domain formulations, are suitable to improve the reliability of the virtual screening ranking of the respective QSAR models. The Optimal Assignment kernel is in most cases the best choice (an example is presented in Figure 1), because it shows comparably good results with each AD formulation and leads to very reliable rankings regardless of how the AD threshold is chosen. The Marginalized Graph kernel seems to be very sensitive to the applicability domain. It is nearly as good as the OAK if the threshold is very restrictive, but the virtual screening performance decreases faster if a looser domain threshold is chosen (Figure 2).

Note that the Figures 1, 2, 3, 4, 5 and 6 cannot be compared with each other by comparing the scores shown on the x-axis because the AD score depends on the AD formulation, the kernel and the data set leading to different score ranges and distributions for each experiment. For comparison purposes, the respective tables (Tables 4, 5, 6) should be taken into account. However, the figures are presented to provide information about the overall behavior of the performance measures regarding all possible threshold choices, and the x-axes share the property that they cover the whole AD range. On the left side, all compounds are retained in the domain, whereas on the right side less than 50 compounds remain. Thus, the general behavior over the complete AD range of the different approaches can be compared to each other using the figures, but not by using a single quantitative threshold evaluation.

### 4.3 Factor Xa

In contrast to the Thrombin experiment, the kernels perform differently in the Factor Xa VS (Table 5). The Optimal Assignment kernel seems to be a good choice, yielding reliable rankings for most of the thresholds (Figure 3). The performance decreases slightly around the -10.0 One-Class threshold score, but recovers rapidly if the threshold is further increased. This result might be caused by the removal of some ligands, due to their low applicability score, which actually had been predicted correctly.

In contrast, the Marginalized Graph kernel does not seem to be able to describe the activity to Factor Xa at all, and the model shows poor generalization ability. Thus, it can be concluded that the applicability domain estimation cannot fix a model by outlier detection, if the model seems not to have learned any relevant relationship between the structures and their activity. This finding also makes a case for the importance of a true external validation of a QSAR model. However, the bad results of the Marginalized Graph kernel, regardless of the choice for the AD threshold, should not be interpreted as a weakness of the applicability domain estimation. If the model itself fails, the AD estimation cannot be expected to fix it.

The most interesting results in the Factor Xa experiment are given by the Flexible OAK (Figure 4). In general, the latter should behave similarly to the Optimal Assignment kernel, but in this case, it shows different results. In contrast to the OAK, the performance of the Flexible OAK does not recover immediately, indicating a more severe cause of the decreased reliability. Instead, this difference might not be caused by the difference in the AD estimation, as the numbers of ligands and decoys above the respective thresholds are nearly identical. Thus, the difference is more likely caused by the different rankings obtained by the QSAR models of OAK and FlexOAK. The OAK produces a good ranking of the innermost 50 compounds (right *x*-axis boundary in Figure 3) yielding an AUROC of nearly 0.8 for the One-Class AD (Figure 3), whereas the FlexOAK leads to an almost arbitrary ranking on the right AD score boundary (Figure 4). A possible cause for this result may be that there are some compounds that are similar enough to the training set to be retained even in the most restrictive applicability domain, but whose SAR is not described correctly by the learned QSAR model. These cases can be seen as example for the second type of error introduced at the beginning of the results section. In this case, the FlexOAK seems to struggle with an activity cliff, but the original Optimal Assignment kernel does not. Therefore, it can be concluded that the FlexOAK overestimates the similarity of molecules regarding their Factor Xa activity (i.e., regards molecules as more similar than they are and thus makes the activity landscape rougher) and that the OAK describes the respective SAR better.

### 4.4 Platelet Derived Growth Factor Receptor β

As in the other screening experiments, the Optimal Assignment kernel shows the best ranking performance regardless of which criterion and applicability threshold are chosen. The Flexible OAK (Figure 5) is competitive in most cases and seems to be able to retain a higher fraction of the ligands in the more stringently defined applicability domains.

The most interesting behavior on this screening data set shows the Marginalized Graph kernel (Figure 6). In the innermost domain presented in Table 6, consisting of the 100 compounds with the highest applicability scores, very few ligands are contained. Thus, regarding this similarity measure, in this case the ligands from the screening set are less similar to the training set than at least 96 (KDE formulation, Table 6) of the decoys. At this point, it is important to keep in mind that the applicability score depends not only on the similarity to the active structures from the training set but also on the inactive ones. This contribution of the inactive training samples may have a bigger impact in the PDGFRβ experiment than in the others, because all kernels regard a much higher proportion of the ligands as outliers than in the other screening experiments (i.e., the decoys might be more similar to the training set (inactives) than the ligands).

In conclusion, this result should not be interpreted as a weakness of the kernel, because it shows good screening performance at the 200 and 100 compound threshold levels. Instead, it should be regarded as the effect of the feature spaces in which the different kernels work and as a hint that the domain should not be defined too strictly.

### 4.5 Significance Test

The application of the AD threshold filter to the screening data sets often has a large impact on the distribution of ligands and decoys as well as on the number of compounds evaluated, and thus changes the probability of getting a certain result by chance. Thus, the improvement of the screening performance in the applicability domain could be a result of the higher probability of getting a good ranking by chance, due to the smaller size and the increased proportion of ligands. To ensure that this situation does not apply and that the improved performance measures are an effect of the removal of outliers rather than the result of an easier task, the statistical significance of the AUROC and the BEDROC scores is calculated using a permutation test.

The permutation test calculates the probability of obtaining an AUROC at least as high as the observed one by a random ranking of the same base distribution (same number of ligands and decoys). The distribution of the AUROC is approximated by generating 10.000 random rankings with the respective numbers of ligands and decoys. The p-value of the observed AUROC is calculated as the relative number of random rankings with an AUROC at least as high as the observed one. Thus, it is an estimate of the probability of producing at least as high an AUROC by chance.

This setup considers the effect of the base distribution on the performance measure of a ranking, and thus allows us to distinguish meaningful rankings from random ones. Note that this significance test does not compare two screening experiments and cannot be interpreted as one experiment being significantly better than the other. It simply ensures that a screening result is not random. The same evaluation was conducted for each of the BEDROC scores.

### 4.6 Choice of the threshold

Unfortunately, our experiments indicate that there seems to be no suitable default threshold for any of the applicability domain estimations. Table 7 shows that the ranges of the threshold, and thus the spatial representations of the applicability domains, strongly depend on the kernel. This dependence alone would be no drawback, but the training data set also affects the possible values of the applicability score. However, there are some guidelines for choosing a threshold that should give good results.

**Table 7:** Ranges of the applicability score for the different combinations of ADE formulation, kernel and experiment

ADE	Kernel	Target	Training Set			Screening Set		
			Min	Max	Avg.	Min	Max	Avg.
KDE	OAK	Thrombin	0.70	0.87	0.82 ± 0.04	0.51	0.77	0.64 ± 0.08
		Factor Xa	0.59	0.75	0.71 ± 0.02	0.47	0.72	0.60 ± 0.08
		PDGFRβ	0.81	0.88	0.85 ± 0.02	0.54	0.70	0.62 ± 0.05
	FlexOAK	Thrombin	0.69	0.86	0.81 ± 0.04	0.54	0.79	0.66 ± 0.08
		Factor Xa	0.60	0.74	0.70 ± 0.02	0.53	0.69	0.61 ± 0.05
		PDGFRβ	0.80	0.88	0.85 ± 0.02	0.54	0.68	0.61 ± 0.04
	MARG	Thrombin	0.81	0.95	0.92 ± 0.03	0.57	0.91	0.74 ± 0.11
		Factor Xa	0.74	0.94	0.91 ± 0.02	0.52	0.88	0.70 ± 0.11
		PDGFRβ	0.92	0.96	0.95 ± 0.01	0.74	0.91	0.82 ± 0.05

wKDE	OAK	Thrombin	0.70	0.87	0.82 ± 0.04	0.51	0.79	0.65 ± 0.09
		Factor Xa	0.59	0.76	0.71 ± 0.02	0.47	0.71	0.59 ± 0.07
		PDGFRβ	0.81	0.88	0.85 ± 0.02	0.54	0.70	0.62 ± 0.05
	FlexOAK	Thrombin	0.69	0.87	0.82 ± 0.04	0.54	0.79	0.66 ± 0.08
		Factor Xa	0.60	0.74	0.71 ± 0.02	0.53	0.70	0.62 ± 0.05
		PDGFRβ	0.80	0.89	0.85 ± 0.02	0.54	0.68	0.61 ± 0.04
	MARG	Thrombin	0.80	0.96	0.93 ± 0.04	0.56	0.93	0.75 ± 0.12
		Factor Xa	0.74	0.94	0.91 ± 0.02	0.52	0.87	0.69 ± 0.11
		PDGFRβ	0.91	0.97	0.95 ± 0.01	0.72	0.91	0.82 ± 0.06

One-Class	OAK	Thrombin	-6.52	1.61	-0.33 ± 1.51	-15.70	-0.79	-8.25 ± 4.65
		Factor Xa	-22.21	4.98	-0.90 ± 3.41	-55.36	-3.94	-29.65 ± 16.01
		PDGFRβ	-1.14	0.92	0.0 ± 0.43	-17.08	-6.63	-11.86 ± 3.24
	FlexOAK	Thrombin	-5.62	1.35	-0.37 ± 2.07	-16.87	-2.42	-9.65 ± 4.50
		Factor Xa	-20.2.0	3.52	-0.96 ± 2.96	-38.80	-3.68	-21.24 ± 10.94
		PDGFRβ	-1.41	0.61	-0.12 ± 0.47	-17.01	-8.46	-12.74 ± 2.66
	MARG	Thrombin	-5.22	1.04	-0.35 ± 1.21	-20.82	-0.03	-10.42 ± 6.45
		Factor Xa	-26.29	2.85	-0.77 ± 3.39	-27.55	0.00	-13.78 ± 8.55
		PDGFRβ	-1.11	0.50	-0.01 ± 0.31	-9.56	-1.37	-5.47 ± 2.54

All applicability domain approaches presented in this work tend to suffer from wrong QSAR model predictions in the innermost applicability domain, which are probably caused by wrong structure-activity relationship assumptions. This impact of possibly wrong QSAR assumptions gets especially apparent in the FlexOAK screening of the Factor Xa data set discussed earlier. Therefore, we propose that the threshold should not be chosen too strictly, because otherwise single mispredictions would have a large impact on the performance. Our experiments indicate that approximately at least 100 compounds should be retained in the domain according to the chosen threshold in order to get performance estimates that are not dominated by a few especially bad predictions. This is also because the BEDROC metric is not robust on data sets of such a small size, because it is designed such that the top ranked (dependent on the choice of α) compounds contribute to 80% of the score. Thus, if only the innermost 100 compounds of the AD are considered, 80% of the BEDROC score for α = 100.0 is based on the first two compounds. Therefore, the BEDROC score for high values of α should not be interpreted too precisely for the more restrictive AD thresholds. However, this effect does not affect the reported statistical significance because the latter considers the increased probability of obtaining a high BEDROC score on a smaller data set.

Another problem that could arise from a too strict threshold can be seen in the PDGFRβ screening using the Marginalized Graph kernel. In the innermost domain, no ligands at all are retained and thus, despite its good performance in less stringent domains, the model is unable to enrich any ligands.

On the other hand, it is obvious that the threshold should not be chosen too loose, in order to remove the clear applicability domain outliers successfully from the prediction.

Finally, the experimental results indicate that in most cases (at least in all of the experiments conducted) the reliability of the virtual screening is improved in the top half and the top third of the screening set according to any of the applicability domain formulations, compared to an evaluation of the whole data set. Thus, instead of defining a default threshold for the AD estimation, the consideration of the lower half of the respective domain as the probable outlier set should be a good rule-of-thumb in most applications. An additional positive effect is that on the respective applicability level, a large proportion of the ligands are usually retained in the domain and the screening reliability is improved. However, the removal of some of the ligands from the screening set due to their low applicability score should not be considered as a drawback of the applicability domain estimation and consideration because these molecules can be expected to be badly predicted otherwise and thus to be neglected because of their low rank regardless.

### 4.7 Comparison of the screening performance inside and outside of the AD

**Figure 7:**
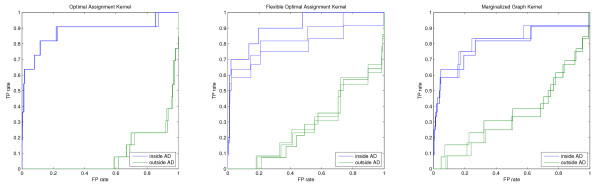
**Comparison of the ROC curves inside and outside the 50% applicability domain for Thrombin**.

**Figure 8:**
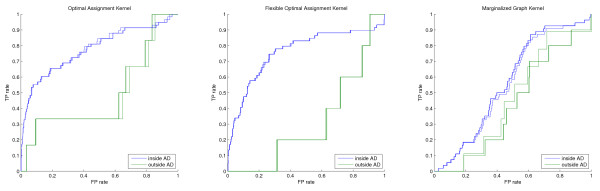
**Comparison of the ROC curves inside and outside the 50% applicability domain for Factor Xa**.

**Figure 9:**
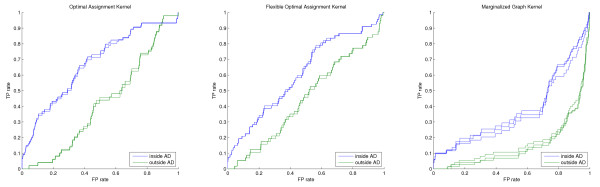
**Comparison of the ROC curves inside and outside the 50% applicability domain for PDGFRβ**.

As discussed earlier the performance of a model in the innermost applicability domain is often not very reliable because, due to the small number of compounds remaining, the influence of single bad predictions is very high. This error can be regarded mostly as the second type of error introduced at the beginning of the results section because a compound has to be very similar to the training samples to be retained in the innermost AD, but obviously has a structure-activity relationship not covered by the model. Thus, to compare the performance gain of restricting the prediction to the molecules, which are regarded as part of the models applicability domain, the threshold should not be chosen too tightly. The comparison of different thresholds showed that in most cases, as discussed in the last section, the performance gain is most robust if half or at least a third of the test samples are retained in the domain. The validity of this choice can be confirmed by comparing the model performance on the molecule sets inside and outside the respective domain. Figures 7, 8 and 9 show the ROC curves inside (blue) and outside (green) for the different screening experiments, kernels, and AD formulations. In all cases, except the top right parts of the OAK and FlexOAK screenings of Factor Xa, the ROC curve inside the domain is better than outside. Thus, even in scenarios in which a model does not give convincing results, as is the case for the FlexOAK and the Marginalized Graph kernel on the PDGFRβ data set, the consideration of the applicability score increases the reliability of the virtual screening.

At this point it should be mentioned that the last case, the Marginalized Graph kernel screen for PDGFRβ ligands, seems to give an inverted ranking, and thus the model does not behave randomly as one would expect for a bad model, and the ROC plot indicates that the performance gets closer to random performance using the 50% threshold. However, the applicability estimation can only consider the fact that for some compounds the model cannot be expected to distinguish ligands from decoys reliably, and not that the ranking outside the domain is truly random. It this case, the model has regarded some decoys as highly active and some ligands as inactive, leading to an inverted ranking. Thus, the random performance of the model inside the domain must be considered as an improvement compared to the inverted ranking outside.

The presented comparisons also indicate that the elimination of some of the active compounds from the screening set should not be regarded as a drawback because the bad rankings obtained on the subset outside the domain can only be the result of these ligands being given low rankings. If only decoys were omitted no sensible ROC curve would have been possible, thus at least some of the ligands must also have been filtered out. Moreover, the ROC curves for the screening of the outside of the applicability domain show that the ranking is at most random. Thus, the ligands outside this 50% domain are in general not predicted to be more active than the decoys and consequently would not have been identified by the model without considering the applicability domain.

Finally, it is important to note that the 50% rule-of-thumb may not always be the best choice. There may be cases in which only the top 10% are predicted reliably, and other cases in which a model is applicable for all compounds. Nevertheless, the experiments show that in general the performance of a model is more reliable in the upper half of the applicability score range than in the lower half, and that this behavior is independent of the real performance of the model.

## 5 Conclusion

The results indicate that the reliability of a virtual screening using structured kernel-based QSAR models can be improved by the identification and removal of compounds that lie outside the applicability domains of the models. To estimate the domain of applicability of a model three formulations were introduced, which can describe the applicability domain of a kernel-based model using only the molecule representation implicitly contained in the kernel.

All formulations were evaluated using three different structured kernels from the literature on three VS experiments. The data sets have been composed from QSAR data sets from the literature for the training of the models and the DUD data sets for virtual screening for the respective targets. Care has been taken that the training and test data sets were completely disjoint.

The conducted experiments show that in general, the performance of the model is considerably better inside the domain than outside, and that this performance gain is not caused by chance due to the changed ligand/decoy ratio. Two of the three AD formulations (Kernel Density Estimation and weighted KDE) can be applied without imposing any computing time overhead because the respective applicability scores can be calculated simultaneously with the prediction using the same iteration. This simultaneous calculation is a big advantage compared to other approaches, which assess prediction reliability and usually need additional iterations.

Unfortunately, the applicability scores computed by the different formulations cannot be directly compared on different experiments, and thus it is not possible to present robust default thresholds to decide whether a compound should be regarded as part of the models domain or not. Despite this lack of a default parameterization, the experiments indicate that omitting the half of the compounds with the lowest applicability score, regardless of which AD formulation and kernel is chosen, improves the reliability of a model considerably and retains a large proportion of the ligands.

An open question, not considered in this work, is whether the AD estimation and the respective partitioning of the screening set influence the chemotype enrichment. This question is interesting and important, but lies outside the scope of this work and will be the subject of further studies.

## Competing interests

The authors declare that they have no competing interests.

## Authors' contributions

NF developed the weighted Kernel Density ADE formulation and implemented all of the presented ADE algorithms. He also wrote most of the text of the article and conducted all experiments. AJ implemented the library for the VS performance measures (AUROC, BEDROC), took part in the experimental design and wrote some sections of the article. GH took part in the experimental design and theoretical development of the presented approaches. AZ helped in the article preparation and proof reading. All authors contributed to the discussing of the results and helped improving the evaluations and conclusions.

## Supplementary Material

Additional file 1 **List of atom and bond descriptors used by the structured kernels**. A complete enumeration of the atom and bond descriptors including their respective references (if applicable) is given in the file. Click here for file

Additional file 2 **Effect of the AD on the VS performance of all combinations of AD, Kernel and Target**. All figures for the AD evaluation of the different experiments are presented in the file. The values for the threshold retaining less than 50 compounds in the respective AD are omitted. Click here for file
