# Supplementary material for: Estimation of the applicability domain of kernel-based machine learning models for virtual screening
Source: J Cheminform. 2010 Mar 11;2:2. doi: 10.1186/1758-2946-2-2 (PMC2851576; doi:10.1186/1758-2946-2-2)
Supplement: Additional file 1 — List of atom and bond descriptors used by the structured kernels. A complete enumeration of the atom and bond descriptors including their respective references (if applicable) is given in the file. [file 1758-2946-2-2-S1.PDF]

Atom and Bond Descriptors used by the Structured Kernels

All descriptors were used as implemented in JOELib2 (https://sourceforge.net/projects/joelib). Please see *http://www.ra.cs.uni-tuebingen.de/software/joelib/tutorial/descriptors/atomProperties.html* for more detailed descriptions. References are only given if citable publications are existent for the respective descriptor.

Atom Descriptors

Nominal Descriptors	Numeric Descriptors
Atom in HB-acceptor (as defined in [1])	Gasteiger-Marsili Partial Charge[2]
Atom in HB-donor (as defined in [1])	Graph Potentials[3]
Atom in donor or acceptor (as defined in [1])	Electrotopological State[4,7]
Atom in terminal carbon	Electrogeometrical State[5,7]
Atom in aromatic system	Atom mass
Atom in ring	Electron Affinity
Atom in conjugated environment [6,7]	Van-der-Waals Volume
Atom is negative	Pauling Electronegativity
Atom is positive	Intrinsic State[5,6]
Atom is chiral	Free electrons count
Atom is axial	Hybridization
	Heavy Valence
	Implicit Valence

Bond Descriptors

Nominal Descriptors	Numeric Descriptors
Bond in aromatic system	Bond length
Bond in ring	
Bond is rotor	
Bond is carbonyl	
Bond is amide	
Bond is primary amide	
Bond is ester	

[1] M. Böhm and G. Klebe, *Development of New Hydrogen-Bond Descriptors and Their Application to Comparative Molecular Field Analyses, J. Med. Chem.*, **2002**, 45, 1585-1597

[2] J. Gasteiger & M. Marsili, A *New Model for Calculating Atomic Charges in Molecules* , *Tetrahedron Lett.*, **1978** , 3181-3184.

[3] W. P. Walters & S. H. Yalkowsky, *ESCHER--A Computer Program for the Determination of External Rotational Symmetry Numbers from Molecular Topology* , *J. Chem. Inf. Comput. Sci.*, **1996** , *36* , 1015-1017

[4] R. Todeschini and V. Consonni, 3-52-29913-0, Wiley-VCH, *Handbook of Molecular Descriptors*.

[5] J. K. Wegner and A. Zell, *Prediction of Aqueous Solubility and Partition Coefficient Optimized by a Genetic Algorithm Based Descriptor Selection Method, J. Chem. Inf. Comput. Sci.*, **2003**, 43, 1077-1084

[6] J. K. Wegner, H. Froehlich, and A. Zell, *Feature Selection for Descriptor based Classification Models. 1. Theory and GA-SEC Algorithm, J. Chem. Inf. Comput. Sci.*, **2004**, 44, 921-930

[7] J. K. Wegner, H. Froehlich, and A. Zell, *Feature Selection for Descriptor based Classification Models. 2. Human Intestinal Absorption*, *J. Chem. Inf. Comput. Sci.*, **2004**, 44, 931-939
