# Supplementary material for: Estimation of the applicability domain of kernel-based machine learning models for virtual screening
Source: J Cheminform. 2010 Mar 11;2:2. doi: 10.1186/1758-2946-2-2 (PMC2851576; doi:10.1186/1758-2946-2-2)
Supplement: Additional file 2 — Effect of the AD on the VS performance of all combinations of AD, Kernel and Target. All figures for the AD evaluation of the different experiments are presented in the file. The values for the threshold retaining less than 50 compounds in the respective AD are omitted. [file 1758-2946-2-2-S2.PDF]

Effect of the AD on the VS performance of all combinations of AD, Kernel and Target

1. Kernel Density AD Estimation

1.1 Thrombin

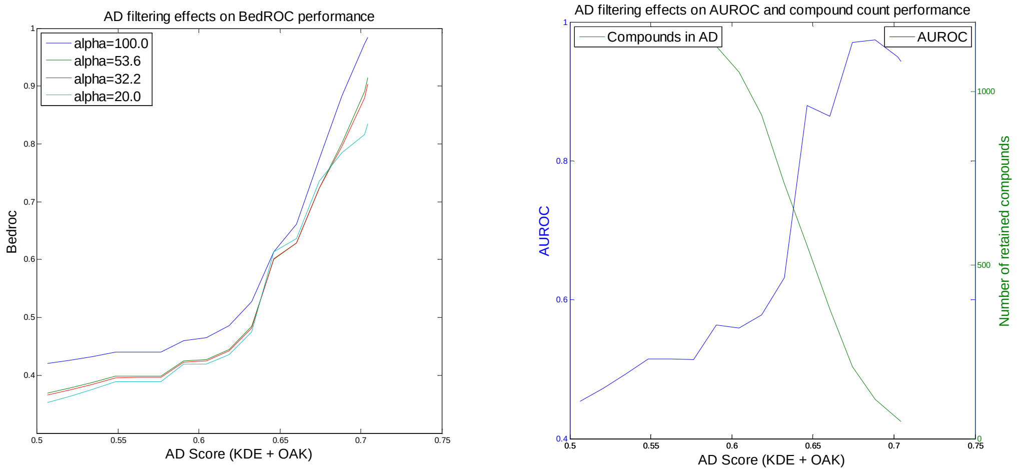

Figure 1: Virtual screening of the Thrombin data set using the Optimal Assignment kernel and the Kernel Density AD Formulation

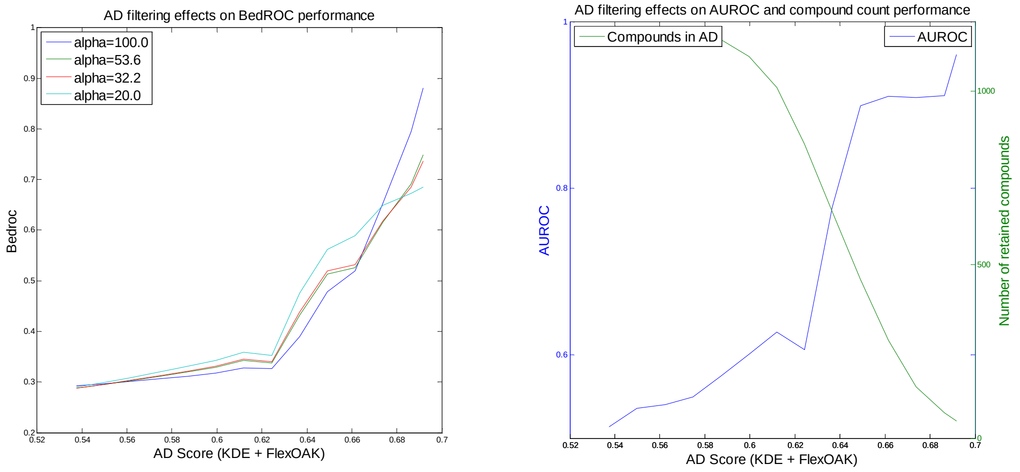

Figure 2: Virtual screening of the Thrombin data set using the FlexOAK kernel and the Kernel Density AD Formulation

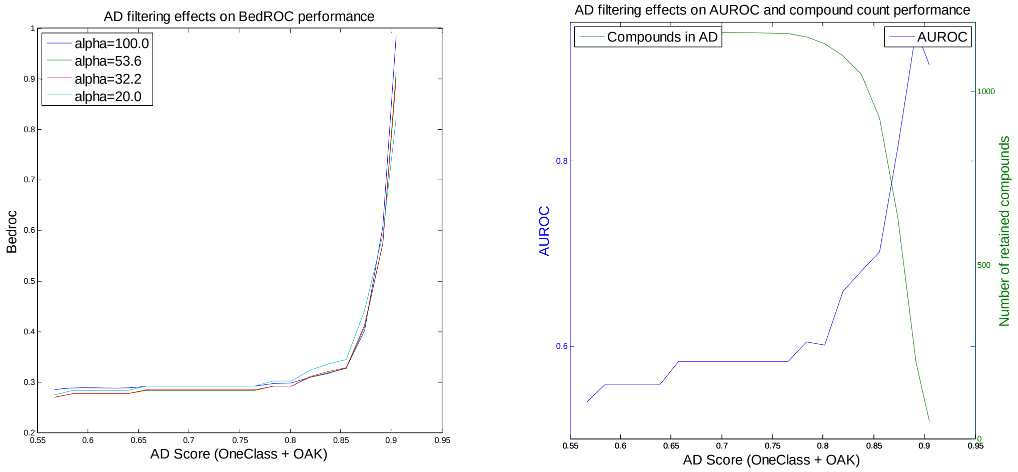

Figure 3: Virtual screening of the Thrombin data set using the Marginalized Graph kernel and the Kernel Density AD Formulation

1.2 Factor Xa

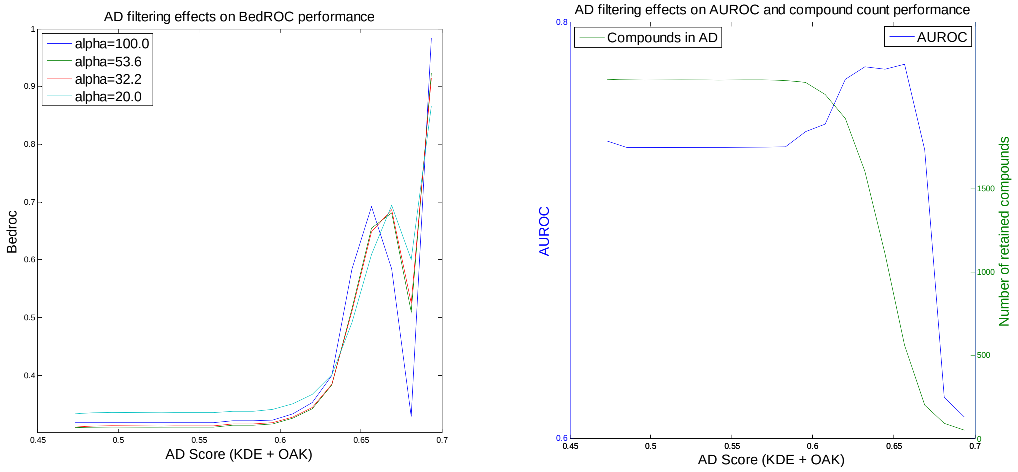

Figure 4: Virtual screening of the Factor Xa data set using the Optimal Assignment kernel and the Kernel Density AD Formulation

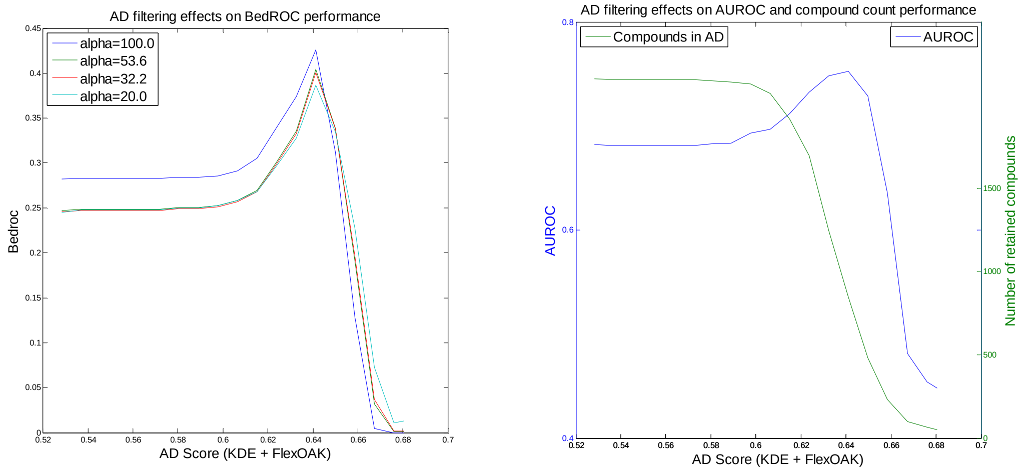

Figure 5: Virtual screening of the Factor Xa data set using the FlexOA kernel and the Kernel Density AD Formulation

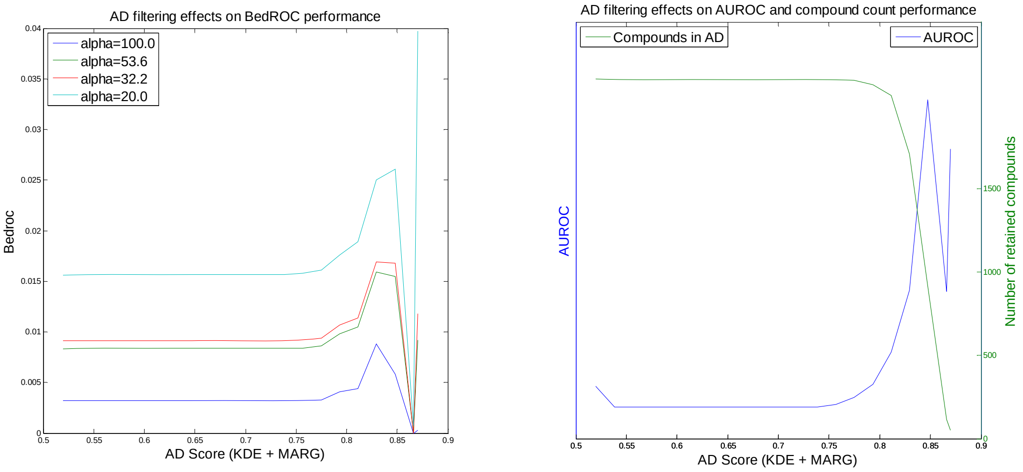

Figure 6: Virtual screening of the Factor Xa data set using the Marginalized Graph kernel and the Kernel Density AD Formulation

1.3 Platelet Derived Growth Factor Receptor β

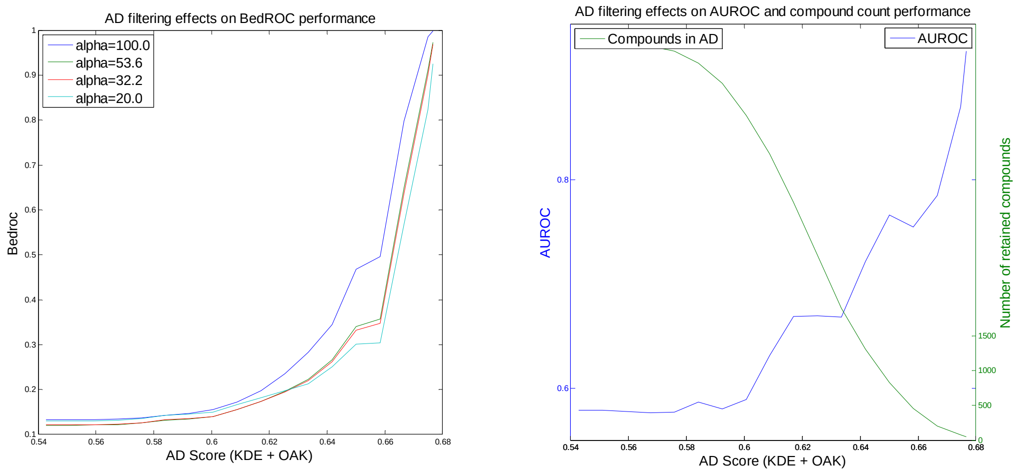

Figure 7: Virtual screening of the PDGFRβ data set using the Optimal Assignment kernel and the Kernel Density AD Formulation

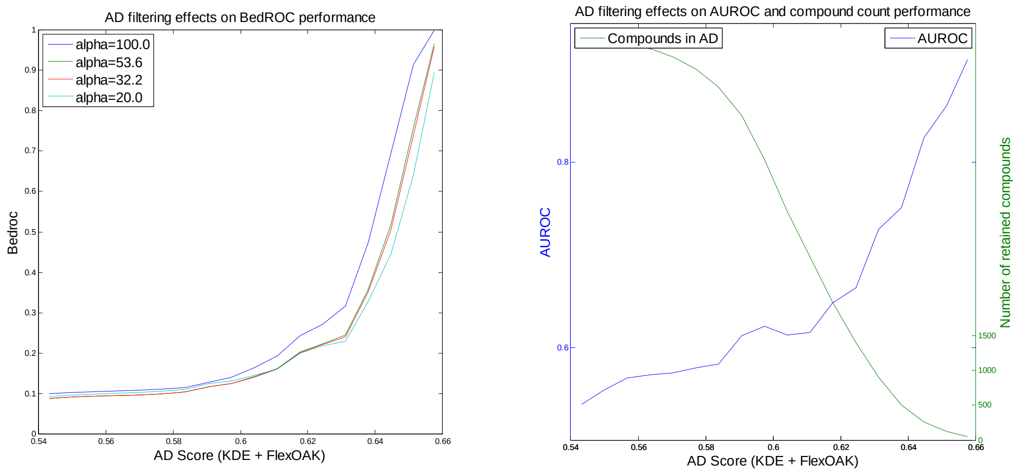

Figure 8: Virtual screening of the PDGFRβ data set using the FlexOAK kernel and the Kernel Density AD Formulation

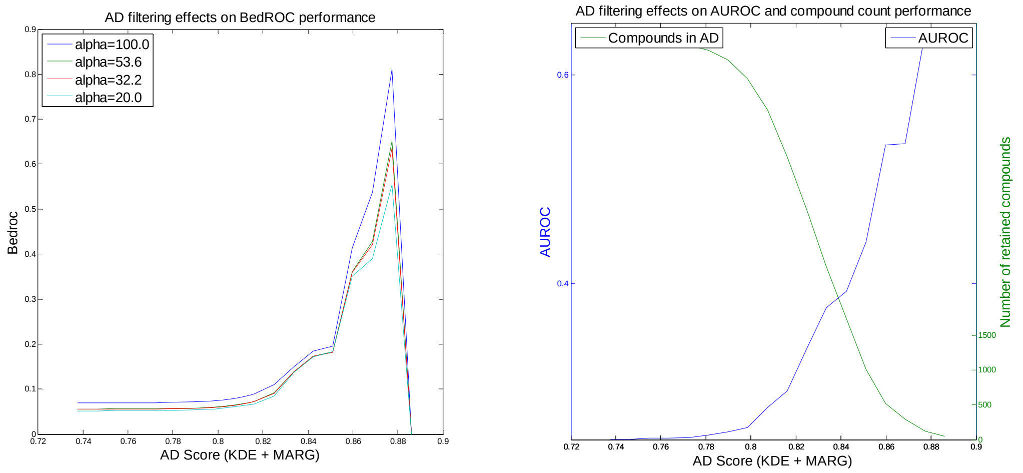

Figure 9: Virtual screening of the PDGFRβ data set using the Marginalized Graph kernel and the Kernel Density AD Formulation

2 Weighted Kernel Density AD Estimation

2.1 Thrombin

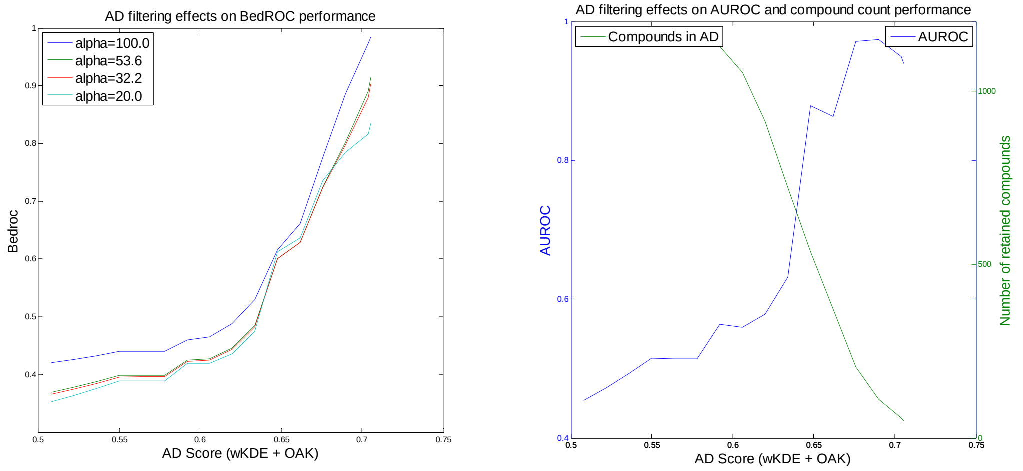

Figure 10: Virtual screening of the Thrombin data set using the Optimal Assignment kernel and the Weighted Kernel Density AD Formulation

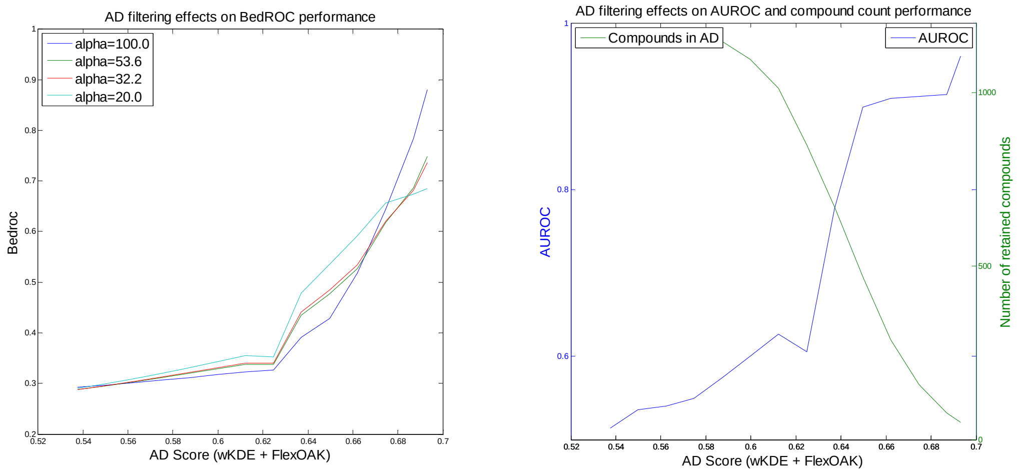

Figure 11: Virtual screening of the Thrombin data set using the FlexOAK kernel and the Weighted Kernel Density AD Formulation

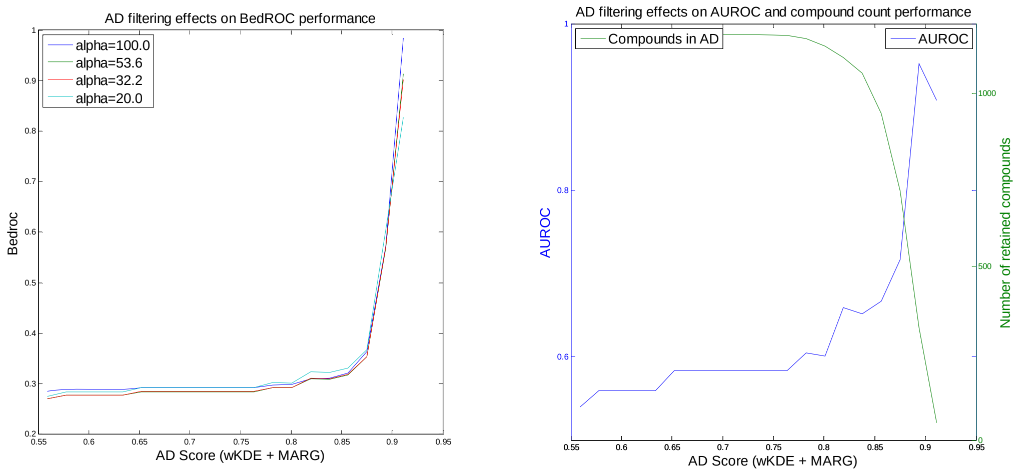

Figure 12: Virtual screening of the Thrombin data set using the Marginalized Graph kernel and the Weighted Kernel Density AD Formulation

2.2 Factor Xa

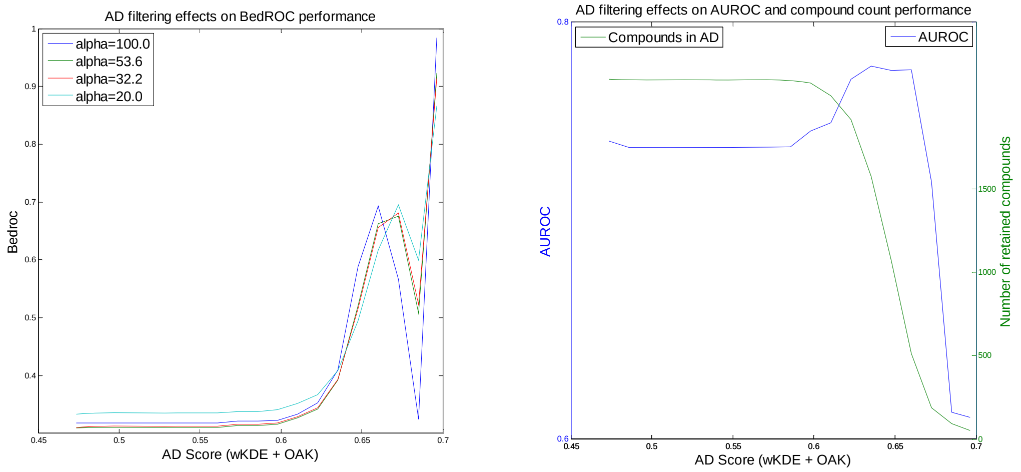

Figure 13: Virtual screening of the Factor Xa data set using the Optimal Assignment kernel and the Weighted Kernel Density AD Formulation

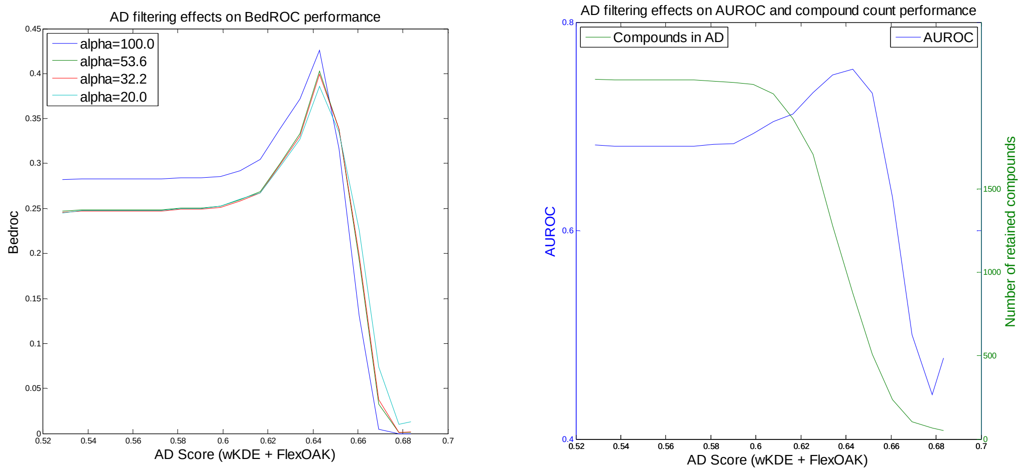

Figure 14: Virtual screening of the Factor Xa data set using the FlexOAK kernel and the Weighted Kernel Density AD Formulation

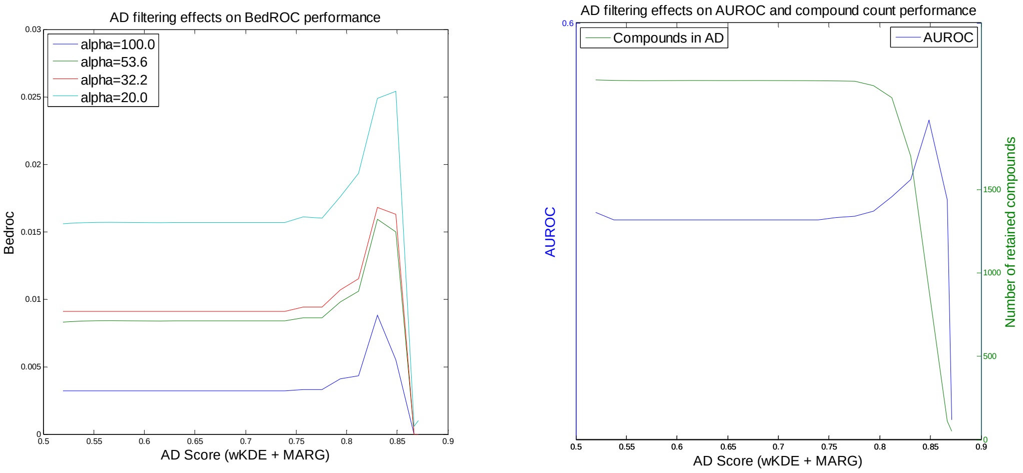

Figure 15: Virtual screening of the Factor Xa data set using the Marginalized Graph kernel and the Weighted Kernel Density AD Formulation

2.3 Platelet Derived Growth Factor Receptor β

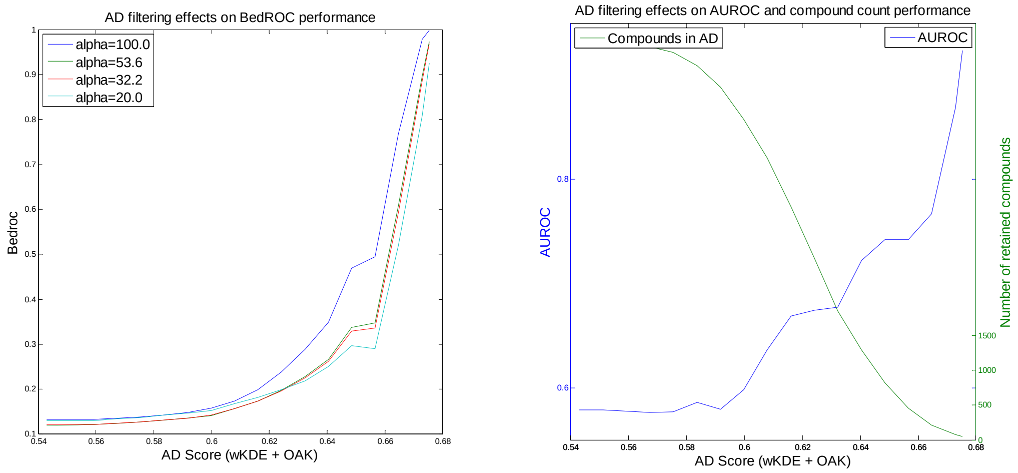

Figure 16: Virtual screening of the PDGFRβ data set using the Optimal Assignment kernel and the Weighted Kernel Density AD Formulation

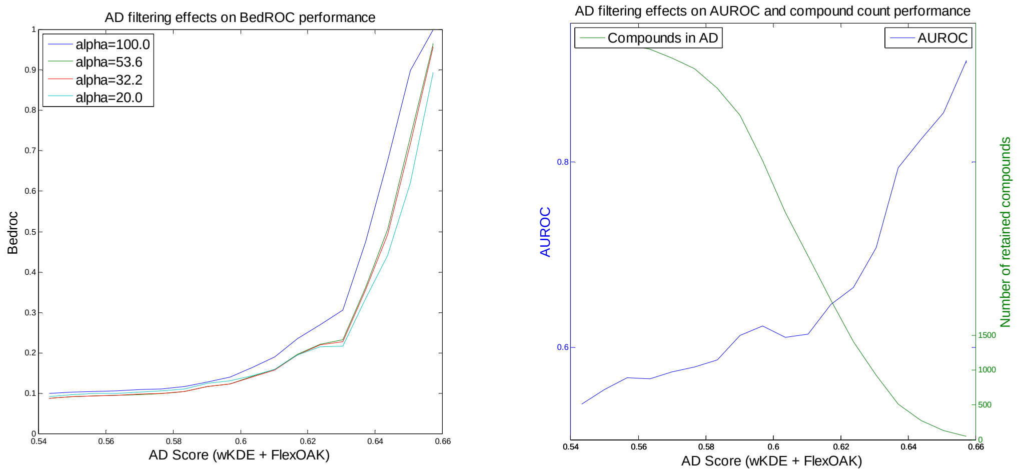

Figure 17: Virtual screening of the PDGFRβ data set using the FlexOAK kernel and the Weighted Kernel Density AD Formulation

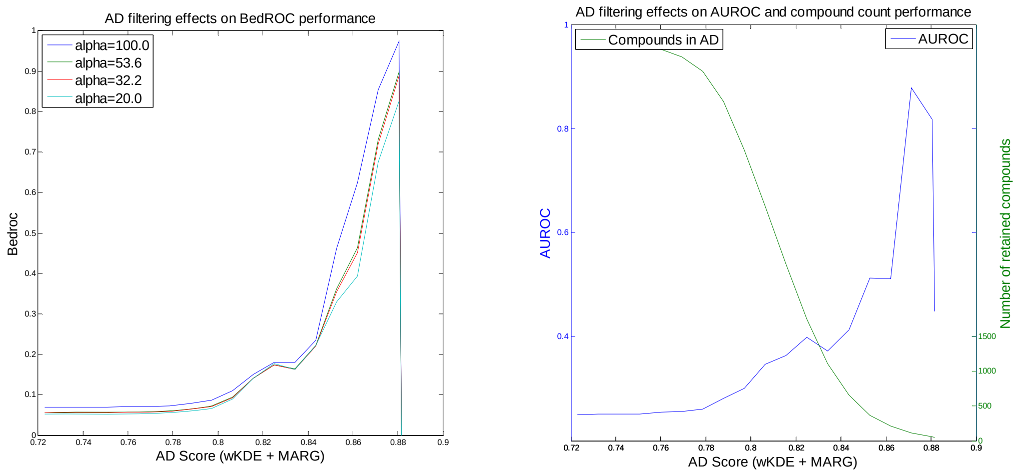

Figure 18: Virtual screening of the PDGFRβ data set using the Marginalized Graph kernel and the Weighted Kernel Density AD Formulation

3 One Class SVM AD Estimation

3.1 Thrombin

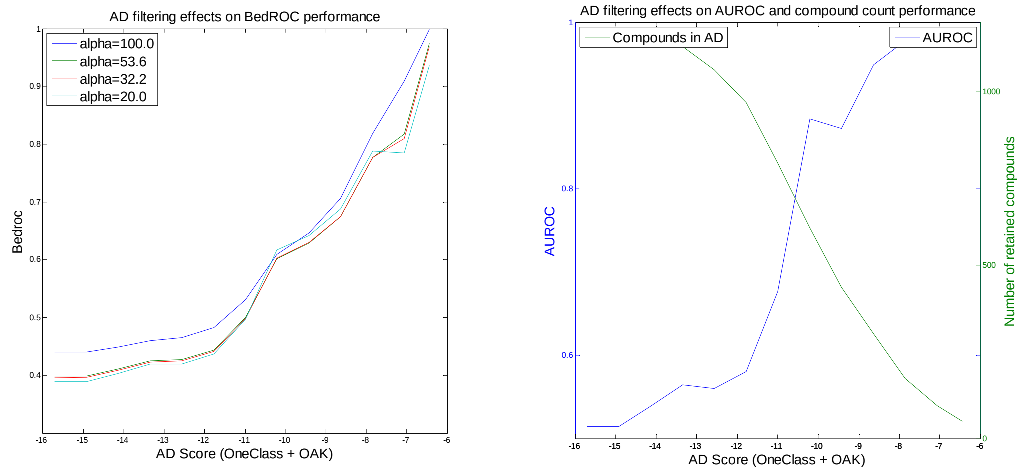

Figure 19: Virtual screening of the Thrombin data set using the Optimal Assignment kernel and the One Class SVM AD Formulation

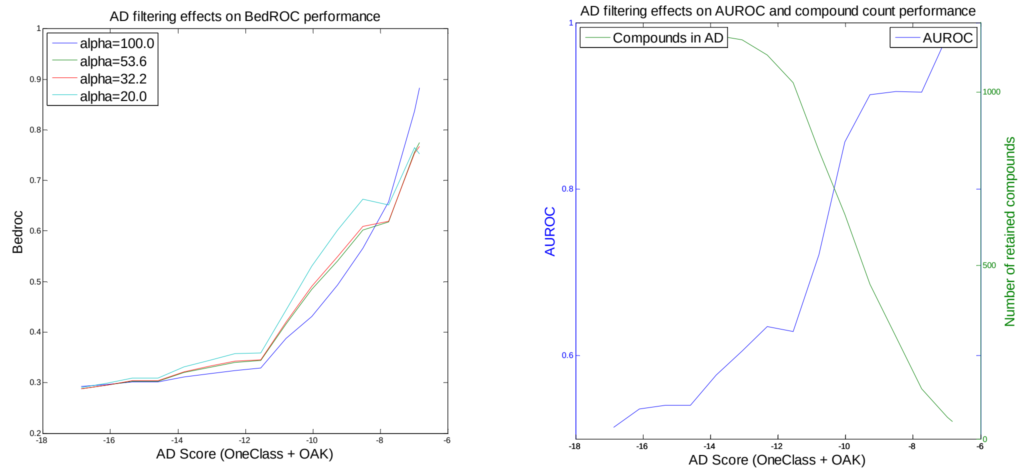

Figure 20: Virtual screening of the Thrombin data set using the FlexOAK kernel and the One Class SVM AD Formulation

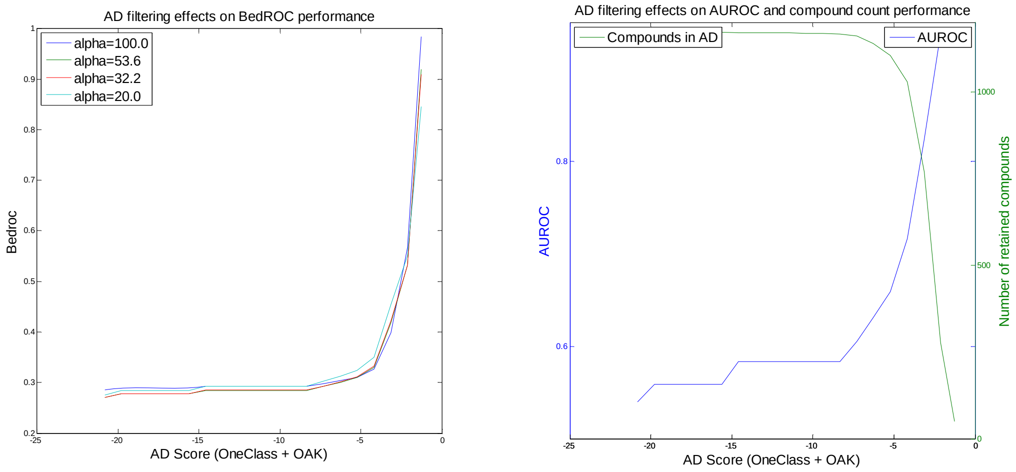

Figure 21: Virtual screening of the Thrombin data set using the Marginalized Graph kernel and the One Class SVM AD Formulation

3.2 Factor Xa

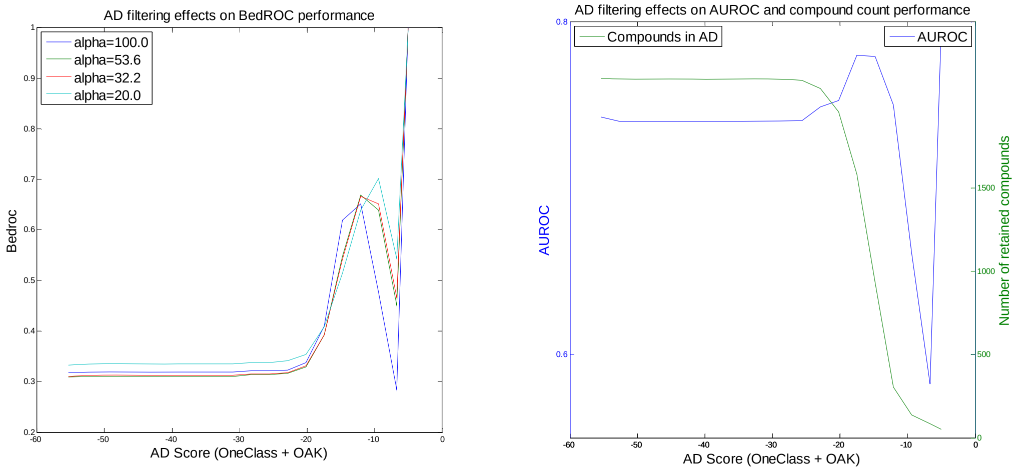

Figure 22: Virtual screening of the Factor Xa data set using the Optimal Assignment kernel and the One Class SVM AD Formulation

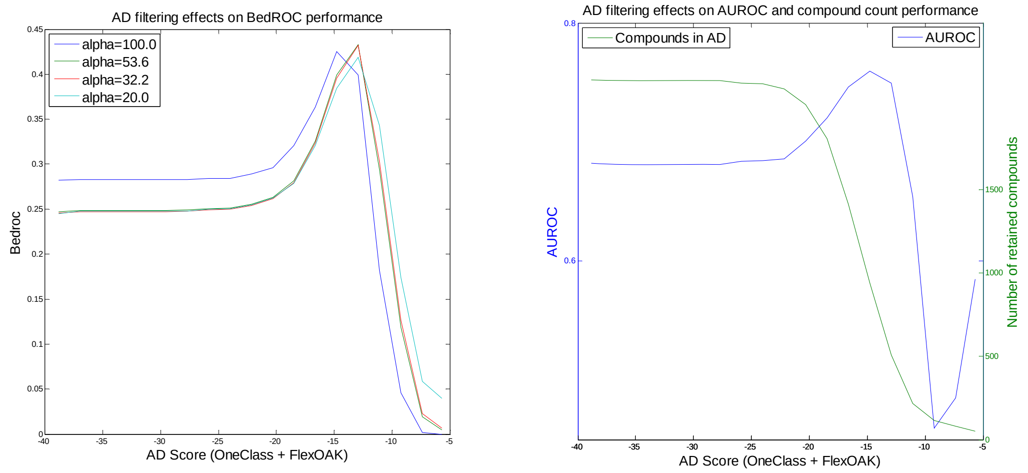

Figure 23: Virtual screening of the Factor Xa data set using the FlexOAK kernel and the One Class SVM AD Formulation

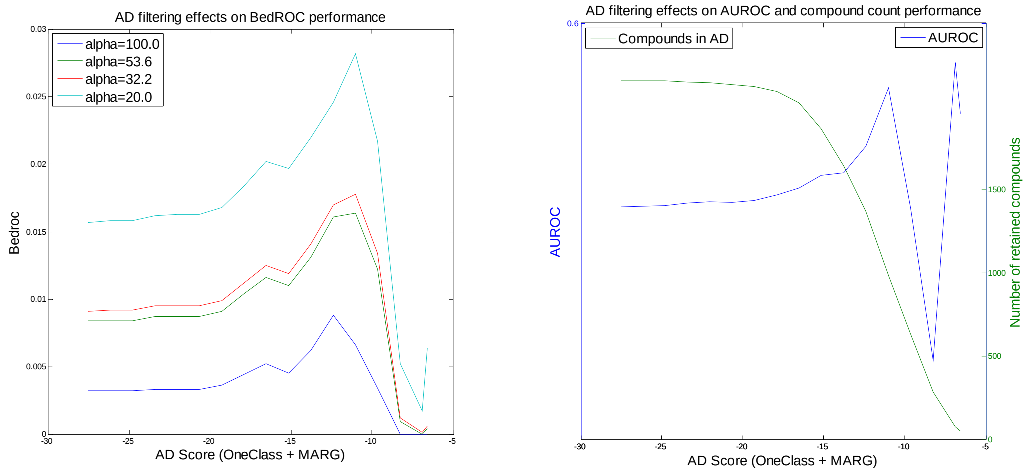

Figure 24: Virtual screening of the Factor Xa data set using the Marginalized Graph kernel and the One Class SVM AD Formulation

3.3 Platelet Derived Growth Factor Receptor β

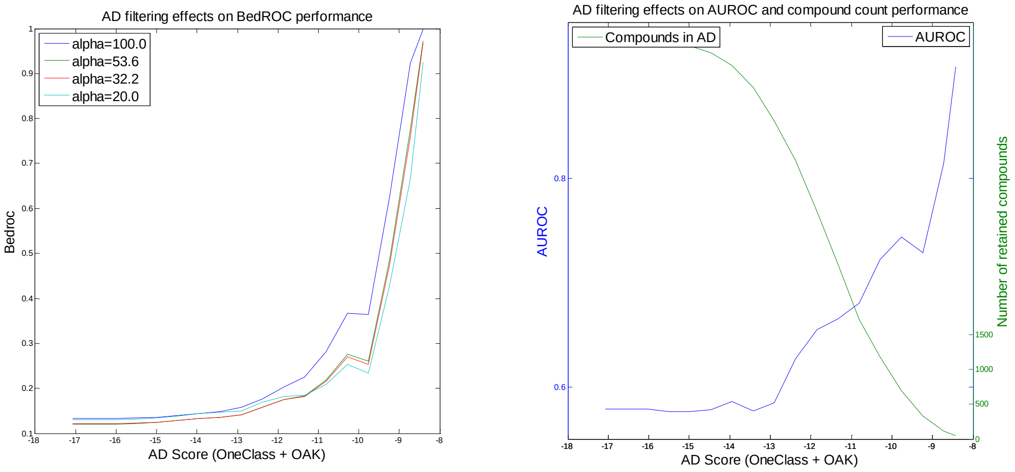

Figure 25: Virtual screening of the PDGFRβ data set using the Optimal Assignment kernel and the One Class SVM AD Formulation

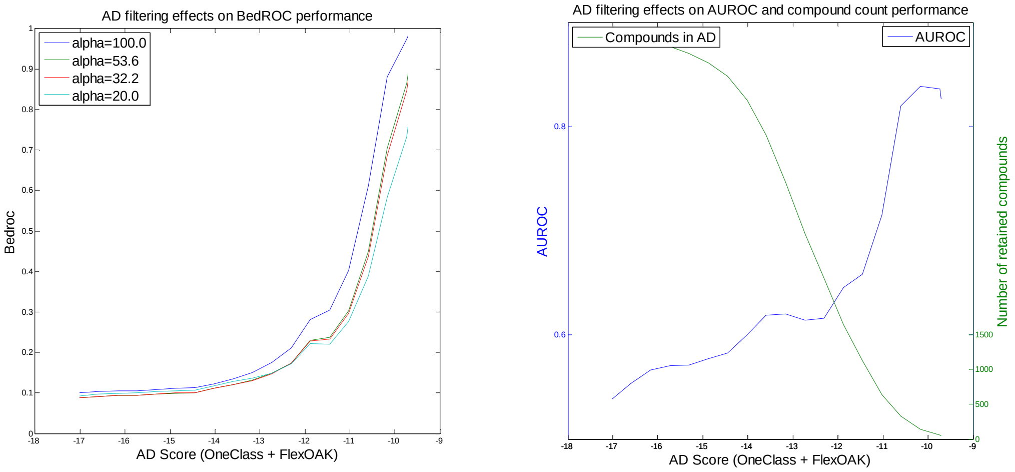

Figure 26: Virtual screening of the PDGFRβ data set using FlexOAK kernel and the One Class SVM AD Formulation

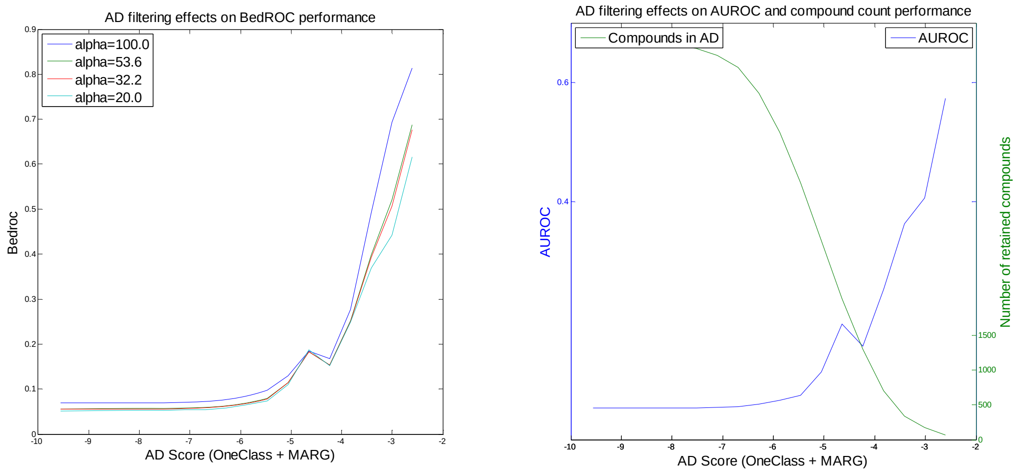

Figure 27: Virtual screening of the PDGFRβ data set using the Marginalized Graph kernel and the One Class SVM AD Formulation
